# l‐Leucine‐Based Layered Coordination Polymer Supports for Immobilizing Basic Salts to Yield Solid CO_2_ Adsorbents Resistant to Moisture and Oxidation

**DOI:** 10.1002/chem.202502886

**Published:** 2026-02-15

**Authors:** Yuki Kohno, Takuji Ikeda, Takashi Makino

**Affiliations:** ^1^ Research Institute for Chemical Process Technology National Institute of Advanced Industrial Science and Technology (AIST) Sendai Miyagi Japan

**Keywords:** amino acid salts, direct air capture (DAC), interlayer spacing, layered coordination polymers, moisture/oxygen resistance

## Abstract

We demonstrate that amino acid‐based coordination polymers can serve as supports for a variety of basic salts. A layered framework, bis(l‐leucinato)zinc(II), Zn(Leu)_2_, prepared by an aqueous, base‐mediated procedure, provides crystallographically defined, carboxylato‐bridged two‐dimensional layered structures with intersheet surfaces. The interlayer spacing is molecularly tunable, and immobilizing potassium l‐leucinate increases the interlayer spacing in two discrete steps. Using this host, diverse basic salts can be immobilized to give solid sorbents that remain macroscopic solids under humid, DAC‐relevant conditions (400 ppm CO_2_, 293 K dew point, 313 K) while delivering equilibrium CO_2_ uptakes of 0.17–1.04 mmol g^−1^. After accelerated oxidative aging in simulated air (400 ppm CO_2_, 21% O_2_, 293 K dew point) at 393 K for 24 h, the amino acid‐based sorbent shows high retention in CO_2_ capacity, whereas a conventional polyamine‐based sorbent exhibits a marked decrease in the capacity. The combination of environmental durability and DAC‐relevant performance establishes Zn(Leu)_2_ as a general and modular platform for sustainable CO_2_ capture technologies.

## Graphical abstract

A layered amino acid–based coordination polymer, (Zn(Leu)_2_), acts as a support that immobilizes diverse basic salts. The resulting solid sorbents remain nondeliquescent under humid, DAC‐relevant feeds while enabling CO_2_ capture. The interlayer‐immobilized sorbents exhibit high oxidative stability after accelerated oxidative aging, unlike conventional polyamine‐based solid sorbents.

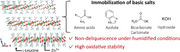

## 1 Introduction

Direct air capture (DAC) technologies, which separate and recover carbon dioxide (CO_2_) directly from the atmosphere, are advancing rapidly worldwide, with early stages of commercial deployment already underway [1]. Among the various DAC approaches, adsorption‐based processes using solid sorbents have attracted considerable attention, as they are generally considered to require less thermal energy for CO_2_ regeneration compared to liquid‐based absorption processes [1].

A wide variety of solid sorbents have been investigated for DAC applications, including polyethyleneimine (PEI) immobilized on porous supports [2, 3, 4, 5], metal organic frameworks (MOFs) [6, 7, 8], porous carbons [9, 10], and covalent organic frameworks (COFs) [11, 12, 13, 14]. While PEI‐based sorbents typically exhibit high CO_2_ uptake at low partial pressures, they suffer from poor oxidative stability, leading to rapid degradation and capacity loss upon exposure to oxygen‐containing streams such as ambient air or flue gas [15, 16, 17, 18]. Furthermore, the oxidative degradation of PEI‐based sorbents can be accelerated by ambient humidity [19]. MOFs and other framework materials offer tunable porosity and chemical functionality but are often limited by high production costs, hydrolytic instability, or performance deterioration under humid conditions [20, 21, 22, 23, 24]. In addition, the majority of these sorbents are derived from fossil‐based resources, highlighting the need for alternative materials that combine stability under realistic DAC conditions with a reduced life‐cycle environmental footprint.

Basic salts such as alkali metal salts of amino acids, which can be derived from renewable feedstocks, have been explored as CO_2_‐reactive materials [25, 26, 27, 28, 29, 30, 31, 32, 33, 34]. These salts react readily with CO_2_, however, they are generally highly hygroscopic and tend to deliquesce upon exposure to humid gas streams [35]. This deliquescence precludes their direct use as solid sorbents for DAC, and consequently, their practical deployment has largely been limited to aqueous solutions in absorption‐based CO_2_ capture systems. To date, studies employing amino acid‐derived materials as solid CO_2_ sorbents have been limited to loading amino acid ionic liquids on porous supports [36] or to modifying amino acids into polymer matrices or MOFs by ion exchange [37, 38].

To deploy basic salts as solid CO_2_ sorbents, designing appropriate support architecture is an effective route to maintain solid‐state integrity under humid streams and to present well‐defined host‐guest environments. Among candidate supports, layered materials are particularly attractive because their interlayer spacing can adapt to the immobilized species [39, 40, 41, 42]. Although layered double hydroxides (LDHs) have been explored as supports for amine‐functionalized sorbents [43, 44, 45], reports remain limited, and the approach is not yet mature. It is also reported that several Zn(II)‐ or Cu(II)‐amino acid coordination polymers form layered or pseudo‐layered frameworks [46, 47, 48, 49]. Building on this precedent, we hypothesized that leveraging amino acid coordination chemistry would provide molecular‐level control over interlayer spacing, surface hydrophobicity, and gas adsorption properties. These features align directly with the requirements of solid‐state CO_2_ sorbents for DAC.

Here, we report a series of humidity‐ and oxygen‐resilient, basic salt‐based solid sorbents for CO_2_ capture that remain in the solid state under exposure to water vapor‐containing CO_2_ streams. The sorbent architecture comprises CO_2_‐reactive basic salts immobilized on zinc(II)‐amino acid coordination polymer supports. Among amino acid ligands, we identify bis(l‐leucinato)zinc(II), Zn(Leu)_2_, as an effective support that hosts a broad range of basic salts. Upon loading, the resulting composites are nondeliquescent under humid CO_2_ and exhibit CO_2_‐capture performance. Moreover, an accelerated durability test revealed that the Zn(Leu)_2_‐based sorbent examined here shows enhanced oxidative stability relative to a benchmark PEI‐based sorbent. We thus deliver a Zn(II)‐amino acid support platform, exemplified by Zn(Leu)_2_, for DAC applications, together with scalable synthetic access to the coordination polymer‐based CO_2_ sorbents.

## 2 Results and Discussion

**FIGURE 1:**
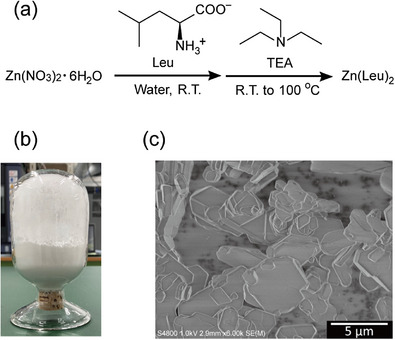
(a) Synthetic route to prepare Zn(Leu)_2_. (b) Photograph of as‐prepared Zn(Leu)_2_ powder. (c) SEM image of Zn(Leu)_2_ showing plate‐like microcrystalline particles (scale bar: 5.0 µm).

An aqueous, base‐mediated route was employed to prepare Zn(Leu)_2_ (Figure 1a). An aqueous solution of Zn(II)(NO_3_)_2_·6H_2_O was prepared, and solid l‐leucine was dispersed into the solution. Triethylamine (TEA) was then added dropwise and stirred at room temperature to deprotonate l‐leucine in situ, giving anionic l‐leucinate that immediately coordinated to Zn(II) to form a milky slurry. To complete the reaction, the suspension was transferred to a sealed pressure vessel and heated to 373 K with stirring. The resulting solid was a white microcrystalline powder, obtainable at laboratory scale in ca. 20 g per batch (Figure 1b).

**FIGURE 2:**
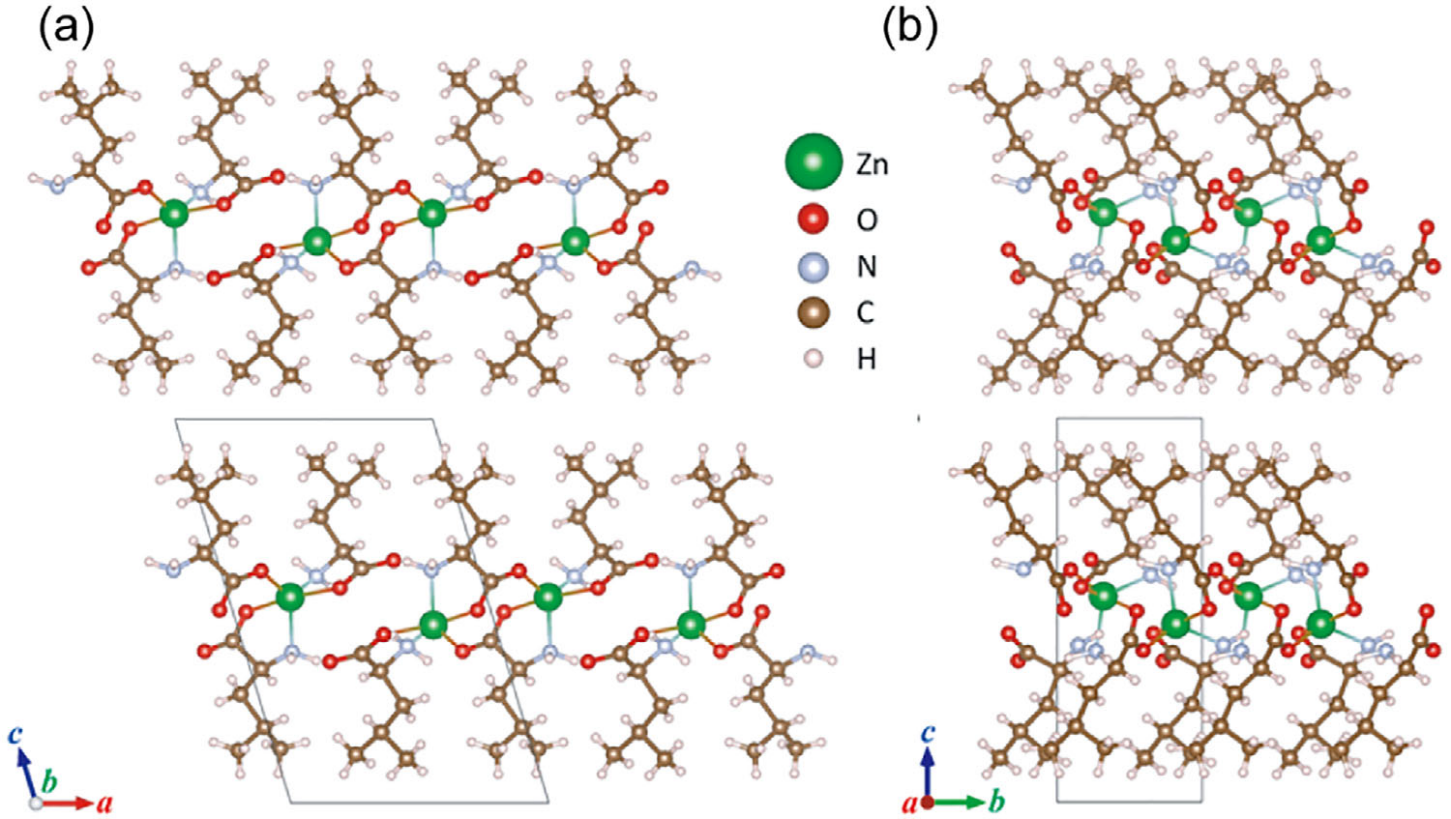
Refined structural models of Zn(Leu)_2_ viewed along (a) [010] and (b) [100] directions, forming a two‐dimensional layered framework structure.

Scanning electron microscopy (SEM) of Zn(Leu)_2_ revealed plate‐like microcrystalline particles (Figure 1c), which are commonly associated with layered solids where anisotropic bonding favors lateral growth. These plate‐like morphologies have been well documented for layered materials [50]. To establish this directly, we determined the crystal structure of Zn(Leu)_2_ by single‐crystal 3D electron diffraction (3D‐ED). The space group and unit cell parameters were determined as monoclinic *P2*_1_, *a* = 9.8 Å, *b* = 5.49 Å, and *c* = 15.2 Å, *β* = 106.7°. Rietveld analysis of PXRD data using the preliminary model obtained by the 3D‐ED analysis yielded an excellent fit, and R‐values converged sufficiently low. (See Supporting Information). Figure 2 shows the crystal structure of Zn(Leu)_2_ with the local coordination environment around Zn(II). Zn(II) centers were five‐coordinate (N_2_O_3_ donor set), each chelated by two bidentate (N,O) l‐leucinato ligands and bound to an additional carboxylato O that bridged to a neighboring unit, generating carboxylato‐bridged two‐dimensional sheets parallel to (001). Within each sheet, N–H···O hydrogen bonds further reinforced the framework, consistent with the layered diffraction signature. Packing views indicated that the hydrophobic l‐leucinato side chains projected from the inorganic–organic backbone into the interlayer region, yielding hydrophobic layer surfaces. The obtained cell parameters and connectivity are congruent with the reported single‐crystal structure of Zn(Leu)_2_ [46]. This fact indicates that the layered structure, which was determined for the first time in a polycrystalline sample, is crystallographically consistent. Furthermore, we measured the N_2_ adsorption isotherms at 77 K for the Zn(Leu)_2_ to analyze the pore structure (See Supporting Information). The Zn(Leu)_2_ exhibited a very low specific surface area of 1.8 m^2^ g^−1^ and a small total pore volume of 8.0 × 10^−3^ cm^3^ g^−1^, indicating that the Zn(Leu)_2_ is essentially nonporous in N_2_ physisorption.

**FIGURE 3:**
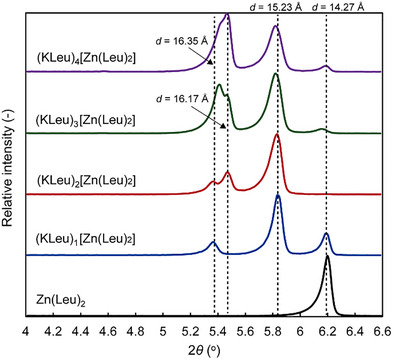
Low‐angle powder x‐ray diffraction (PXRD) patterns of Zn(Leu)_2_ and (KLeu)_*n*_[Zn(Leu)_2_] (*n* = 1–4).

Loading potassium l‐leucinate (KLeu) onto Zn(Leu)_2_ was carried out by the wet impregnation method using ethanol to give (KLeu)_*n*_[Zn(Leu)_2_] (*n* = 1–4), where *n* denotes the molar ratio of KLeu to the Zn(Leu)_2_ support. The low‐angle PXRD of (KLeu)_*n*_[Zn(Leu)_2_] revealed a two‐step expansion of the interlayer spacing with increasing KLeu loading (Figure 3). Pristine Zn(Leu)_2_ showed a basal reflection at 2*θ* = 6.20°, corresponding to *d*_001_ = 14.27 Å. Upon initial impregnation, a new basal reflection emerged at 2*θ* = 5.80° (*d*_001_ = 15.23 Å) while the 6.20° peak diminished (Stage I). At higher loadings, the basal plane peak shifted further and developed at 2*θ* = 5.40–5.46° (*d*_001_ = 16.17–16.35 Å; Stage II). We define the interlayer spacing as *d*_2_ = *d*_001_ − *d*_1_, where *d*_1_ is the intralayer thickness estimated from the refined structural model (*d*_1_ = 12.59 Å). With this definition, *d*_2_ increased in two discrete steps from ∼1.65 Å (pristine) to ∼2.64 Å (Stage I) and then to ∼3.58–3.76 Å (Stage II). The discrete shifts indicated at least two preferred interlayer spacings upon KLeu insertion. In Stage II, the low‐angle profile was broad and asymmetric, implying coexisting interlayer spacings or a distribution thereof, rather than a single, well‐defined basal family. It should be noted that the expansion of the interlayer spacing was observed in other basic salts beyond KLeu. As shown in Figure S6, we measured PXRD data for a series of guest‐loaded samples, (KSar)_1_[Zn(Leu)_2_], (KBzIm)_1_[Zn(Leu)_2_], and (K_2_CO_3_)_1_[Zn(Leu)_2_] (structures of the immobilized salts are shown in Table 1). The PXRD patterns for the samples showed the characteristic basal reflections of the Zn(Leu)_2_ host. In addition, new basal reflections were observed relative to pristine Zn(Leu)_2_, consistent with guest‐dependent changes in interlayer spacing.

**TABLE 1:** Molecular structures of the basic salts and the CO_2_ uptake of the corresponding solid sorbents measured at 400 ppm CO_2_, 293 K dew point, 313 K. Sorbents are denoted as (KX)_1_[Zn(Leu)_2_] for monovalent anions (1‐8, 11‐14, 16), and (K_2_Y)_1_[Zn(Leu)_2_] for divalent anions (9, 10, 15).

	Adsorbents	Structure of basic salt guests	CO_2_ adsorbed / mmol g^−1^
1	(KGly)_1_[Zn(Leu)_2_]	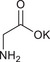	0.36
2	(KAla)_1_[Zn(Leu)_2_]	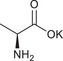	0.51
3	(KVal)_1_[Zn(Leu)_2_]	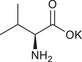	0.44
4	(KLeu)_1_[Zn(Leu)_2_]	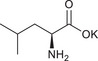	0.47
5	(KIle)_1_[Zn(Leu)_2_]	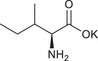	0.29
6	(KPhe)_1_[Zn(Leu)_2_]	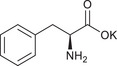	0.31
7	(KHis)_1_[Zn(Leu)_2_]	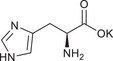	0.17
8	(KPro)_1_[Zn(Leu)_2_]	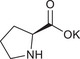	0.42
9	(K_2_Asp)_1_[Zn(Leu)_2_]	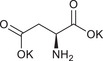	0.51
10	(K_2_Glu)_1_[Zn(Leu)_2_]	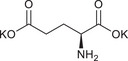	0.35
11	(KTau)_1_[Zn(Leu)_2_]	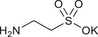	0.63
12	(KSar)_1_[Zn(Leu)_2_]	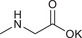	0.84
13	(KBzIm)_1_[Zn(Leu)_2_]	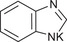	0.66
14	(KHCO_3_)_1_[Zn(Leu)_2_]	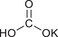	0.48
15	(K_2_CO_3_)_1_[Zn(Leu)_2_]	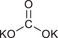	1.04
16	(KOH)_1_[Zn(Leu)_2_]	KOH	0.54

The CO_2_ uptake for (KLeu)_1_Zn(Leu)_2_ was evaluated to determine the experimental conditions (See Supporting Information). As a preparatory step to determine the pretreatment conditions, (KLeu)_1_Zn(Leu)_2_ was heated to 393 K under humid N_2_ with a dew point of 293 K to desorb CO_2_, cooled to 313 K, and then exposed to 400 ppm CO_2_ in N_2_ at the same dew point to determine the gravimetric CO_2_ uptake (mmol g^−1^). The first uptake was 0.99 mmol g^−1^. The sample was then desorbed at 413 K under humid N_2_ and cooled again to 313 K. The second uptake decreased to 0.48 mmol g^−1^, and subsequent adsorption and desorption cycling retained this capacity over at least four cycles under the same conditions (Figure S8). To explore the CO_2_ uptake mechanism, Diffuse Reflectance Infrared Fourier Transform spectroscopy (DRIFTS) was performed for the CO_2_‐adsorbed (KLeu)_1_Zn(Leu)_2_. After the first CO_2_ adsorption, bands were observed in regions typically associated with bicarbonate (i.e., a band at 1,026 cm^−1^ for bending vibrations COH group in bicarbonate) [51] and with proton transfer at amino groups to form NH_3_^+^ (i.e., a band at ∼2130 cm^−1^ for combination band of the NH_3_^+^ asymmetric bending and twisting vibrations) [52], and these features weakened upon heating to 393 K under reduced pressure below 100 Pa (Figure S9). After the second adsorption, bands attributable to bicarbonate were not evident (Figure S10). Taken together, these observations suggest a change in the CO_2_ reaction pathway between the first and later exposures, possibly from one that involves bicarbonate formation to one in which bicarbonate is not produced, for example, carbamate species with lower amine efficiency. Guided by these preliminary results, the second cycle uptake was used for comparisons in this study.

The CO_2_ uptake of (KLeu)_*n*_[Zn(Leu)_2_] (*n* = 1–4) under DAC‐relevant conditions was evaluated. Figure S11 plots CO_2_ uptake as a function of the loading amount of KLeu per mol of Zn(Leu)_2_ (*n*:1) and shows an approximately linear increase over the loading range explored. The sorbents remained as macroscopic solids after CO_2_ adsorption, with no evidence of liquid exudation/deliquescence. A comparison with reported PEI‐supported sorbents is summarized in Table S4, indicating that (KLeu)_*n*_[Zn(Leu)_2_] achieves CO_2_ uptakes comparable to PEI‐based benchmarks under DAC conditions.

To map the scope of immobilizable bases, we prepared a series of Zn(Leu)_2_‐supported sorbents impregnated with basic salts (1–16), including aliphatic and aromatic amino acid salts, acidic and basic amino acid salts, amino acid derivatives, azole salt, and simple hydroxide, carbonate, and bicarbonate salts. The photographs in Figure S12 show that all samples remained macroscopic solids after gas exposure with the same CO_2_/H_2_O/N_2_ composition as described above, indicating that immobilization within the layered host suppresses the deliquescence typically associated with these salts. As a control experiment, bulk KGly powder was exposed to the same humid test gas, and deliquescence occurred (Figure S13), whereas its immobilized counterpart remained solid. Table 1 shows the molecular structures of salts (1–16) and the gravimetric CO_2_ uptake of the corresponding sorbents, showing that the Zn(Leu)_2_ host accommodates a wide variety of basic salts and enables them to function as solid CO_2_ sorbents. We also examined sorbents based on other amino acid coordination polymer supports. Under the same CO_2_ uptake test, (KLeu)_1_[Zn(Leu)_2_] delivered the highest gravimetric CO_2_ uptake, whereas (KGly)_1_[Zn(Gly)_2_] and (KAla)_1_[Zn(Ala)_2_] were markedly lower, each below 0.10 mmol g^−1^ (Figure S30).

Across the α‐amino acid salts (1–10), CO_2_ uptake ranged from 0.17 to 0.51 mmol g^−1^. When the CO_2_ uptake was normalized to the moles of amino acid salts and expressed as the “base efficiency (mol‐CO_2_/mol‐base)”, the lowest value was observed for KHis (0.09), whereas the highest was found for K_2_Asp (0.27). Although materials with higher basicity are generally known to show greater CO_2_ uptake, the relationship between the p*K*_b_ value of the amino acids and the base efficiency revealed no clear correlation (Figure S31). Rather than invoking a single property (e.g., basicity of salts), multiple factors are likely to govern the CO_2_ uptake, including guest orientation/packing and occupancy within the interlayer spacing. These aspects will be addressed in future work.

**FIGURE 4:**
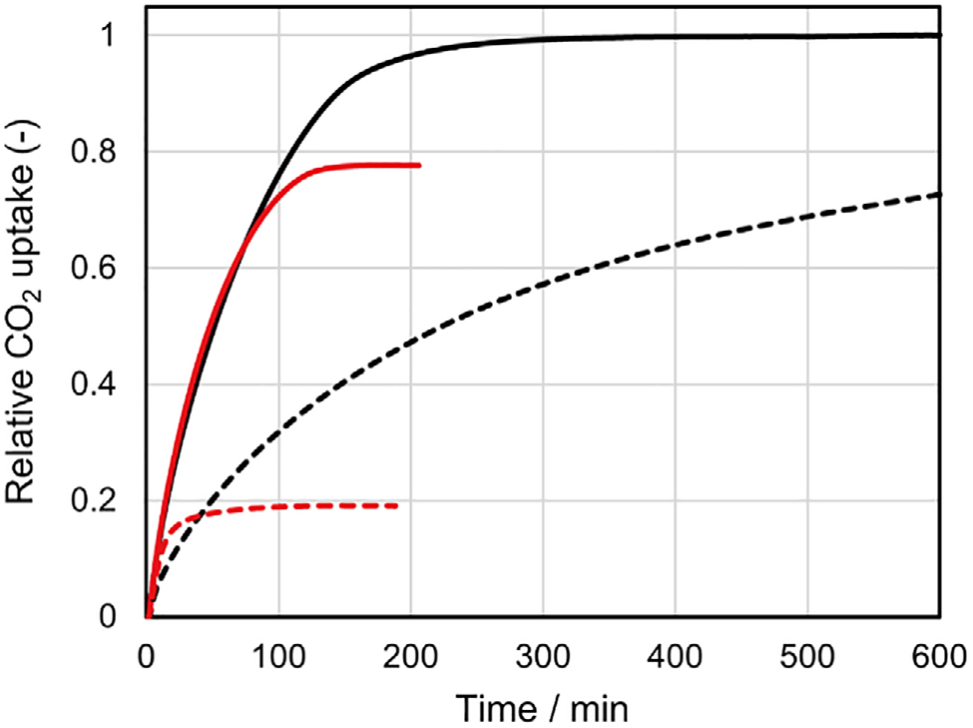
Time courses of relative CO_2_ uptake values for (KLeu)_1_[Zn(Leu)_2_] (solid line) and PEI@Silica (dotted line), before (black) and after (red) the accelerated oxidative aging. CO_2_ uptake is normalized to the equilibrium sorption value before the degradation.

To prove oxidative stability, an accelerated durability test was conducted for (KLeu)_1_[Zn(Leu)_2_] and a benchmark polyamine‐based sorbent with 50 wt% PEI impregnated into porous silica support, denoted as PEI@Silica. Both sorbents were exposed to simulated air (400 ppm CO_2_, 21% O_2_ in N_2_; dew point 293 K) at 393 K for 24 h for accelerated oxidative aging, followed by CO_2_ uptake measurements under the same conditions described above. In both cases, CO_2_ uptake values are normalized to each sorbent's equilibrium values before degradation (= 1.0) to highlight relative retention (Figure 4). (KLeu)_1_[Zn(Leu)_2_] largely retained the CO_2_ capacity (78% retention), whereas PEI@Silica exhibited a pronounced decrease (19% retention). These results indicate that immobilized amino acid salts within Zn(Leu)_2_ host are substantially more resistant to oxidative degradation than a representative PEI‐based benchmark, consistent with the oxygen sensitivity of polyamine‐based sorbents noted in literature. It should be noted that the accelerated durability test for KLeu alone was also conducted, and 64% retention of the CO_2_ uptake was observed (Figure S32), suggesting that immobilization within the interlayer spacing of Zn(Leu)_2_ enhances oxidative stability.

Elemental (CHN) analysis corroborates this interpretation. For (KLeu)_1_[Zn(Leu)_2_], the nitrogen content was essentially retained (from 8.46 to 8.08 wt%, 96% retention) with only a minor change in molar N/C ratio (from 0.167 to 0.162). In contrast, PEI@Silica lost nitrogen substantially (from 11.80 to 7.42 wt%, 63% retention) with a concomitant drop in N/C ratio (from 0.536 to 0.435) (Table 2), consistent with oxidative loss of amine functionality. Thus, nitrogen retention in the amino acid–based system mirrors its preserved CO_2_ capacity, whereas nitrogen depletion in the PEI system aligns with oxygen‐induced performance degradation under the same test.

**TABLE 2:** Elemental analysis (CHN/wt%) of (KLeu)_1_[Zn(Leu)_2_] and PEI@Silica before and after the accelerated oxidative aging. Values in parentheses indicate the retention (%) of each element after aging.

Adsorbents	Accelerated oxidative aging	Elemental analysis		
		C/wt%	H/wt%	N/wt%
(KLeu)_1_[Zn(Leu)_2_]	Before	43.55	7.21	8.46
	After	42.81	7.09	8.08
		(98%)	(98%)	(96%)
PEI@Silica	Before	18.89	4.81	11.80
	After	14.64	2.47	7.42
		(76%)	(51%)	(63%)

We acknowledge that the CO_2_ uptakes obtained in this work (0.17–1.04 mmol g^−1^ at 400 ppm CO_2_) are lower than those reported for some state‐of‐the‐art solid DAC adsorbents. For example, a recently reported polyamine‐immobilized COF (COF‐999) achieved 0.96 mmol g‐1 under dry and 2.05 mmol g^−1^ under humid conditions, both measured at 400 ppm CO_2_, together with excellent cycling stability [11]. At the same time, recent studies on MOF‐based physisorbents highlighted that tailoring the pore environment can mitigate competitive H_2_O adsorption and thereby improve CO_2_ adsorption under humidified conditions [53, 54]. In light of this context, we emphasize that the key contribution of the present study is the introduction of a layered Zn(Leu)_2_ host platform that can immobilize diverse basic salts while maintaining macroscopic solid‐state integrity under humid, DAC‐relevant conditions and showing enhanced oxidative stability. The present host‐guest platform, therefore, provides a distinct materials space for further optimization of guest chemistry and practical applications under realistic operating conditions.

## 3 Conclusion

We established Zn(Leu)_2_ as a layered host platform for humidity‐resilient solid CO_2_ sorbents. An aqueous base‐mediated synthesis furnished a microcrystalline solid. The 3D‐ED measurement for Zn(Leu)_2_ confirmed carboxylate‐bridged two‐dimensional sheets with intersheet surfaces. The interlayer spacing is molecularly tunable, and immobilizing KLeu expanded *d*_001_ from 14.27 Å to 15.23 Å (Stage I) and 16.17–16.35 Å (Stage II). Under DAC‐relevant conditions (400 ppm CO_2_, 293 K dew point, 313 K), immobilization of chemically diverse basic salts yielded nondeliquescent solids with equilibrium CO_2_ uptakes spanning 0.17–1.04 mmol g^−1^ across 16 guests. Notably, after accelerated oxidative aging under simulated air at 393 K for 24 h, (KLeu)_1_[Zn(Leu)_2_] retained 78% of its CO_2_ capacity with 96% nitrogen retention, whereas PEI@Silica retained only 19% with 63% nitrogen retention. Together, these results present a general and modular route to solid‐state, humidity‐ and oxidation‐resilient CO_2_ sorbents in which interlayer chemistry and spacing can be engineered by guest selection. Ongoing work advances the fundamental chemistry of CO_2_ capture in the interlayer environment. It moves toward practical applications through sorbent manufacturing by granulation and optimization of DAC process conditions for demonstration and deployment.

## Conflicts of Interest

The authors declare no conflicts of interest.

## Supporting information



Experimental procedures for the aqueous base‐mediated synthesis of Zn(Leu)_2_ and preparation of the corresponding solid CO_2_ sorbents by the solvent impregnation method, details of CO_2_ uptake measurements under DAC‐relevant conditions, methods for 3D‐ED and other measurements, and additional figures and tables are provided.
