# Supplementary material for: l‐Leucine‐Based Layered Coordination Polymer Supports for Immobilizing Basic Salts to Yield Solid CO2 Adsorbents Resistant to Moisture and Oxidation
Source: Chemistry. 2026 Feb 15;32(12):e02886. doi: 10.1002/chem.202502886 (PMC13037347; doi:10.1002/chem.202502886)
Supplement: Supplementary file 1 — Experimental procedures for the aqueous base‐mediated synthesis of Zn(Leu)2 and preparation of the corresponding solid CO2 sorbents by the solvent impregnation method, details of CO2 uptake measurements under DAC‐relevant conditions, methods for 3D‐ED and other measurements, and additional figures and tables are provided. [file CHEM-32-e02886-s001.docx]

l-Leucine-Based Layered Coordination Polymer Supports for Immobilizing Basic Salts to Yield Solid CO2 Adsorbents Resistant to Moisture and Oxidation

Yuki Kohno,*[a] Takuji Ikeda,[a] and Takashi Makino[a]

[a] Dr. Y. Kohno, Dr. T. Ikeda, Dr. T. Makino
Research Institute for Chemical Process Technology
National Institute of Advanced Industrial Science and Technology (AIST)
4-2-1, Nigatake, Miyagino-ku, Sendai, Miyagi 983-8551, Japan
E-mail: yuki-kouno@aist.go.jp

# Table of Contents

1. Materials and Methods

2. Synthesis and Sample Preparation

3. Characterization

4. Crystal Structure Analysis

5. CO2 Uptake Measurements

6. Accelerated durability Test

7. Additional Figures and Tables

8. References in Supporting Information

# 1. Materials and Methods

l-Glycine (Gly, >99.0%), l-alanine (Ala, >99.0%), l-valine (Val, 98.0%), l-leucine (Leu, >99.0%), l-isoleucine (Ile, >98.0%), l-phenylalanine (Phe, >98.0%), l-histidine (His, >99.0%), l-proline (Pro, >99.0%), l-aspartic acid (Asp, >99.0%), l-glutamic acid (Glu, >99.0%), taurine (Tau, >98.0%), sarcosine (Sar, >98.0%), and benzimidazole (BzIm, >98.0%) were purchased from Tokyo Chemical Industry Co. Zinc(II) nitrate hexahydrate (Zn(NO3)2·6H2O, >99.0%), triethylamine (TEA, >99.0%), potassium bicarbonate (>99.5%), potassium carbonate (>99.5%), and 50% potassium hydroxide solution were purchased from FUJIFILM Wako Pure Chemical Co. Branched polyethyleneimine (PEI, number-average molecular weight of 800) and porous silica (Davisil, pore size 60 Å, particle size 35–60 mesh) were purchased from Sigma-Aldrich. Other chemicals and solvents were used as received.

Elemental analysis was performed with a vario MICRO cube (Elementar, Germany) instrument. Diffuse Reflectance Infrared Fourier Transform spectroscopy (DRIFTS) was performed with Nicolet iS50R (ThermoFisher Scientific, USA). Scanning electron microscopy (SEM) was performed with S-4800 (Hitachi High-Tech, Japan).

# 2. Synthesis and Sample Preparation

**2-1. Preparation of amino acid-based coordination polymers**

Amino acid coordination polymers were prepared via an aqueous, base-mediated procedure. As a representative example, bis(l-leucinato)zinc(II), Zn(Leu)2, was prepared as follows: an aqueous solution of Zn(NO3)2·6H2O was prepared, to which Leu and TEA were added with stirring (molar ratio of Leu:TEA:Zn(NO3)2·6H2O = 2:2:1). After stirring at room temperature, the mixture was transferred to a capped, pressure-resistant vessel and maintained at 373 K with stirring for 2 h. The vessel was cooled to room temperature, and the resulting solid and mother liquid were transferred to an Erlenmeyer flask. The solid was washed with ethanol and collected by membrane filtration. The product was dried *in vacuo* at 353 K to afford Zn(Leu)2.

**Zn(Leu)2**: White powder, 92% yield, Anal. Calcd. for C12H24N2O4Zn: C, 44.25; H, 7.43; N, 8.60. Found: C, 43.85; H, 7.54; N, 8.51.

**Zn(Gly)2**: The coordination polymer was synthesized analogously to the procedure for Zn(Leu)2 except that Gly was used in place of Leu. White powder, 91% yield. Anal. Calcd. for C4H8N2O4Zn: C, 22.50; H, 3.78; N, 13.12. Found: C, 22.26; H, 4.19; N, 12.92.

**Zn(Ala)2**: The coordination polymer was synthesized analogously to the procedure for Zn(Leu)2 except that Ala was used in place of Leu. White powder, 75% yield. Anal. Calcd. for C6H12N2O4Zn: C, 29.83; H, 5.01; N, 11.60. Found: C, 30.16; H, 5.03; N, 11.46.

**2-2. Impregnation of basic salts into amino acid-based coordination polymers**

Loading basic salts onto amino acid-based coordination polymers was carried out by wet impregnation method using ethanol or ethanol/water mixture as solvents. As a representative, (KLeu)1[Zn(Leu)2], in which an equimolar amount of KLeu was immobilized on Zn(Leu)2, was prepared as follows: In a vial, 50% aqueous KOH, Leu, and ethanol (solvent) were combined at a 1:1 molar ratio of KOH:Leu. The mixture was stirred at room temperature until Leu dissolved, giving a solution of KLeu. Zn(Leu)2 support was then added at a 1:1 molar ratio of KLeu : Zn(Leu)2, and the suspension was stirred at room temperature for 2 h. The solvent was removed by a rotary evaporator, and the residue was dried *in vacuo* at 393 K (<10 Pa) to afford the CO2 adsorbent (KLeu)1[Zn(Leu)2].

**(KLeu)*n*[Zn(Leu)2] (*n* = 1–4)**: Following the procedure described above, the CO2 sorbents were prepared analogously by setting the molar ratio of KLeu:Zn(Leu)2 to n:1.

**(KGly)1[Zn(Leu)2]:** The CO2 sorbent was prepared analogously to the representative procedure, except that Gly was used in place of Leu.

**(KAla)1[Zn(Leu)2]:** The CO2 sorbent was prepared analogously to the representative procedure, except that Ala was used in place of Leu.

**(KVal)1[Zn(Leu)2]:** The CO2 sorbent was prepared analogously to the representative procedure, except that Val was used in place of Leu.

**(KIle)1[Zn(Leu)2]:** The CO2 sorbent was prepared analogously to the representative procedure, except that Ile was used in place of Leu.

**(KPhe)1[Zn(Leu)2]:** The CO2 sorbent was prepared analogously to the representative procedure, except that Phe was used in place of Leu.

**(KHis)1[Zn(Leu)2]:** The CO2 sorbent was prepared analogously to the representative procedure, except that His was used in place of Leu, and ethanol/water (4:1 v/v) mixture was used as the solvent.

**(KPro)1[Zn(Leu)2]:** The CO2 sorbent was prepared analogously to the representative procedure, except that Pro was used in place of Leu.

**(K2Asp)1[Zn(Leu)2]:** The CO2 sorbent was prepared analogously to the representative procedure, except that Asp was used in place of Leu, employing a 2:1:1 molar ratio of KOH:Asp:Zn(Leu)2 with ethanol/water (2:1 v/v) mixture as the solvent.

**(K2Glu)1[Zn(Leu)2]:** The CO2 sorbent was prepared analogously to the representative procedure, except that Glu was used in place of Leu, employing a 2:1:1 molar ratio of KOH:Glu:Zn(Leu)2 with ethanol/water (2:1 v/v) mixture as the solvent.

**(KTau)1[Zn(Leu)2]:** The CO2 sorbent was prepared analogously to the representative procedure, except that Tau was used in place of Leu, and ethanol/water (4:1 v/v) mixture as the solvent.

**(KSar)1[Zn(Leu)2]:** The CO2 sorbent was prepared analogously to the representative procedure, except that Sar was used in place of Leu.

**(KBzIm)1[Zn(Leu)2]:** The CO2 sorbent was prepared analogously to the representative procedure, except that BzIm was used in place of Leu.

**(KHCO3)1[Zn(Leu)2]:** The CO2 sorbent was prepared analogously to the representative procedure, except that KHCO3 was used in place of KOH/Leu, employing a 1:1 molar ratio of KHCO3:Zn(Leu)2, with ethanol/water (1:1 v/v) mixture as the solvent. The solvent was removed by heating the solution at 393 K, instead of using a rotary evaporator.

**(K2CO3)1[Zn(Leu)2]:** The CO2 sorbent was prepared analogously to the representative procedure, except that K2CO3 was used in place of KOH/Leu with a 1:1 molar ratio of K2CO3:Zn(Leu)2. Ethanol/water (1:1 v/v) mixture was used as the solvent, and removed by heating at 393 K instead of using a rotary evaporator.

**(KOH)1[Zn(Leu)2]:** The CO2 sorbent was prepared analogously to the representative procedure, except that only KOH was used with a 1:1 molar ratio of KOH:Zn(Leu)2.

**(KGly)1[Zn(Gly)2]:** The CO2 sorbent was prepared analogously to the representative procedure, except that Gly and Zn(Gly)2 were used in place of Leu and Zn(Leu)2.

**(KAla)1[Zn(Ala)2]:** The CO2 sorbent was prepared analogously to the representative procedure, except that Ala and Zn(Ala)2 were used in place of Leu and Zn(Leu)2.

**2-3. Impregnation of polyethyleneimine into porous silica support**

PEI-impregnated porous silica was prepared by wet impregnation method as follows: In a vial, PEI, porous silica, and ethanol were combined and stirred at room temperature for 2 h, using a 0.5:1 mass ratio of PEI:silica. The solvent was removed by a rotary evaporator, and the residue was dried *in vacuo* at 353 K (<10 Pa) to afford PEI@Silica.

# 3. Characterization

**3.1. Solid-state MAS NMR**

The local environments of 13C and 15N nuclei were investigated utilizing a solid-state magic-angle spinning nuclear magnetic resonance (MAS NMR) experiment. All MAS NMR spectra were collected on an AVANCEIII 400 WB (*B*0 = 9.4 T, BrukerBioSpin, Japan) spectrometer using a 3.2 mm HXY CP/MAS probe operated at 100.621 MHz for 13C, and 40.551 MHz for 15N.

The {1H}–13C cross-polarization (CP)/MAS NMR spectrum (Figure S1) was measured at a spinning frequency of 8 kHz with a contact time of 4000 ms, a cycle delay time of 4 s, and the SPINAL64 1H decoupling sequence. The {1H}–15N cross-polarization CP/MAS NMR spectrum (Figure S2) was also measured at a spinning frequency of 8 kHz with a contact time of 1500 ms, a cycle delay time of 10 s, and the SPINAL64 1H decoupling sequence.

The 13C and 15N chemical shifts were referenced to adamantane solid, and 15N-enriched NH4Cl, respectively, as an external standard.

**3.2. Powder X-ray diffraction**

The prepared powder samples were sealed into a borosilicate glass capillary tube with an inner diameter of 0.5 mm. Accurate PXRD data were collected on the D8 Advance with Vario-1 diffractometer (Bruker AXS, Japan) in a modified Debye-Scherrer geometry with a Ge(111) incident monochromator operated at 45 kV–50 mA at room temperature using Cu Kα1 radiation (*λ* = 0.1540593 nm). The diffractometer is equipped with a 1D position-sensitive detector (VÅNTEC-1) with a coverage 2*q* angle of 6°. The µr (µ: linear absorption coefficient; *r*: sample radius) values of each sample tube, estimated through transmittance measurement, were less than 1.0, so X-ray absorption correction was omitted.

**3.3. N2 adsorption measurement**

N2 adsorption measurements were carried out at 77 K using a BELSORP MAX volumetric adsorption analyzer (MicrotracBEL, Japan). Prior to measurement, the Zn(Leu)2 sample was degassed by evacuation at 393 K for 30 h. The final pressure before the adsorption measurement was 1.5 × 10-5 Pa. Adsorption isotherms were collected at 77 K (liquid N2 bath), and the specific surface area was calculated using the Brunauer-Emmett-Teller (BET) method. The total pore volume was estimated at *P/P*0 = 0.99.

# 4. Crystal Structure Analysis

**4.1. 3D-ED analysis**

An initial crystal structure of Zn(Leu)2 was solved from electron diffraction data collected on an XtaLAB Synergy-ED (Rigaku, Co. Ltd. Japan) [s1]. The shutterless data were collected using a continuous crystal rotation method (3D-ED) with a 658.6 mm camera length. The electron beam was operated at 200 kV using a LaB6 cathode, and ED images in 775 × 365 pixels with a pixel size of 100 mm × 100 mm were collected on a high-speed, high-sensitivity photon-counting detector (HyPix-ED). The dataset contains 200 frames, saved and processed (CrysAlisPro for ED v171.43.144) with a goniometer scan step of 0.5°, and the total electron beam exposure time was 110 s. The completeness of the collected data was 66  % at *d* > 0.9 Å, which was sufficient to get a packing structure of Zn(Leu)2 using intrinsic phasing by the direct method SHELXT (Sheldrick, 2015) [s2,s3] and refined by full-matrix least-squares technique on |*Fobs*|2 using the Olex2 1.7 [s4] software package. The experimental conditions and analytical information are summarized in Table S1. A selected microcrystalline of Zn(Leu)2 exhibiting a thin platy morphology and its reciprocal 3D-ED maps are shown in Figure S3.

**4.2. Rietveld analysis**

Initially, the lattice constants and the space group derived from systematic absences were verified by the indexing analysis using a program conograph [s5]. The constructed initial models in this way were modified and refined in detail by the Rietveld method with various geometrical constraints, which gave the final structure model. Rietveld refinement of PXRD data was collected by the following using the versatile RIETAN-FP v3.0 package [s6]. Electron density distribution was calculated from structure factors, *F*obs, obtained by the Rietveld refinement by the maximum entropy method (MEM) (i.e. the MEM/Rietveld method [s7] using the program ERIS [s8]. Crystal structure models and MEM electron density maps were visualized by the program VESTA3 [s9].

In the indexing analysis, lattice constants and space group candidate were *a =* 9.588 Å, *b* = 5.390 Å, *c* = 14.892 Å, *b* = 106.832°, and *P*21 or *P*21/*m*. The obtained lattice constants were introduced as initial values for the following Rietveld analysis.

In the Rietveld refinement, fractional coordinates (*x*, *y*, *z*) and isotropic atomic displacement parameters (*B*iso) of the total number for all 43 independent sites were refined. An 11th-Legendre background function, a modified split pseudo-Voigt profile function, and the partial profile relaxation technique [s10] were adopted. Interatomic distances, *d*, of C–C, C–N, C–O, C–H, and N–H bonds were constrained to be 1.54 ± 0.02 Å, 1.48 Å ± 0.01 Å, 1.40 Å ± 0.01 Å, 0.95 Å ± 0.01 Å, and 1.02 Å ± 0.01 Å, respectively. Additionally, bond angles, *f*, of C–C–C, C–C–H, O–C–O, C–C–N, C–N–H, H–N–H and N–C–H were constrained to be 109.47° ± 2.0°, 109.47° ± 2.0°, 120.0° ± 2.0°, 109.47° ± 2.0°, 107.5° ± 2.0°, 107.0° ± 2.0°, and 106.5° ± 2.0°. The *B*iso for the O sites were constrained to be equal: *B*(O1a) = *B*(O1b) = *B*(O2a) = *B*(O2b). Additionally, linear constraint conditions were set assuming that the isotropic atomic displacement parameters are equal for all sites of the same element. After refinement, the refined chemical composition was estimated as C12H24N2O4Zn (*Z* = 2), which coincided completely with that of the previous report [46]. The refined structural models are represented in Figure 2. The *R* factors were decreased to sufficiently low levels (Figure S4, Table S2). Structural parameters *g*, *x*, *y*, *z*, and *B*iso of Zn(Leu) obtained by the structural refinement are listed in Table S3 in accordance with the Crystallographic Information Files (CIF) form.

In addition, two reliability factors, *RF* and *wRF*, obtained by the MEM electron density analysis were decreased by 0.74 % and 1.30 %, respectively. Not only the covalent bonds within the l-leucine molecules, but also the covalent-like weak bonding electrons between Zn and l-leucine were clearly visualised. No localized electron density inconsistent with the structural model was observed at all (Figure S5).

# 5. CO2 Uptake Measurements

A schematic illustration of the CO2 uptake apparatus is shown in Figure S7. The setup comprised mass flow controllers (MFCs) for N2, CO2, and O2 (KOFLOC, 3660, Japan or HORIBA STEC, ESC E40, Japan), an aluminum block heater capable of stirring and independently controlling the temperature of each test tube (EYELA, PPS-5511, Japan), two humidification tubes (no. 1 and no. 2), one sorbent tube for CO2 uptake (no. 4), and buffer tubes placed upstream and downstream of the sorbent tube (no. 3 and no. 5). The system was further equipped with a mass flow meter (MFM) for gas-flow measurement (KOFLOC, 3760, Japan), a data logger for flow recording (GRAPHTEC, GL240, Japan), gas analyzers for CO2 and H2O concentrations (LI-COR, LI-850, United States), O2 sensor (Mischel Instruments, Microx 122 with OC-39 sensor, United Kingdom), and a PC for data collection of the CO2/H2O concentration. The CO2 uptake measurements were performed as follows: the rationale for adopting this procedure is discussed in the main text.

**Step 1 (Mixed gas preparation and CO2 adsorption):** Under N2 atmosphere, ~100 mg of sorbent was charged into tube no. 4 and mounted on the apparatus. Tubes no. 4 and no. 5 were set to 313 K. Pure water was placed in the humidification tubes (no. 1 and no. 2), and tubes no. 1, no. 2, and no. 3 were maintained at 293 K. The mixed gas supplied from the CO2/N2 MFCs was passed through the humidification tubes and directed to the CO2/H2O analyzers via a bypass line. The MFC setpoints and humidifier temperatures were adjusted to achieve 400 ppm CO2 and a dew point of 293 K. The CO2/H2O/N2 mixture was then fed to the sorbent tube (no. 4) to initiate adsorption. Equilibrium CO2 uptake was determined when the outlet CO₂ concentration returned to 400 ppm ± 2 ppm, at which point the gas supply to the sorbent was stopped. Possible liquefaction of the sorbent upon exposure to humidified gas was visually checked.

**Step 2 (Desorption):** N2 gas was passed through the humidification tubes to prepare an N2/H2O mixture with a dew point of 293 K, which was then fed to tube no. 4 while heating the tube to 413 K to desorb CO2. Desorption was deemed complete when the outlet CO2 concentration decreased to < 5 ppm, after which the sorbent tube was cooled to 313 K.

**Step 3 (CO2 uptake measurement):** As in Step 1, a CO2/H2O/N2 mixture at 400 ppm CO2 and a dew point of 293 K was prepared and fed to the sorbent tube (no. 4). Gas flow and CO2/H2O concentrations were recorded from the start of dosing. The equilibrium adsorption was determined as in step 1, and the measurement was terminated.

CO2 uptake in the sorbents was calculated using the equation below:

Here, *n* is the CO2 uptake (mmol g-1). *P* the pressure (Pa), *F*0 the gas flow rate (L min-1), *R* the gas constant (Pa L mol-1 K-1), *T* the temperature (K), m the sample mass (g), *c*in(*t*) the CO2 concentration at the inlet at time *t* (ppm), and *c*out(*t*) the CO2 concentration at the outlet at time *t* (ppm). Unless stated otherwise, *P* = 101325 Pa, *R* = 8.314 × 103 Pa L mol-1 K-1, and *T* = 273 K were used. *F*0 was taken as the mean flow rate of the conditioned feed gas during the adsorption step, measured with a mass flow meter and logged every 2 seconds. Because the decrease in flow rate associated with CO2 uptake was negligible, the average over the entire adsorption period was used as *F*0. *c*in(*t*) was taken as the average CO2 concentration of the outlet gas after equilibrium had been reached in Step 3 (*i.e.*, the baseline feed concentration). *c*out(*t*) denotes the CO2 concentration of the outlet gas at time *t* as measured by the gas analyzer.

# 6. Accelerated durability Test

The accelerated durability test to exposure simulated air into (KLeu)1[Zn(Leu)2] or PEI@Silica at elevated temperature was carried out with the same apparatus to measure CO2 uptake as described in Chapter 5.

**Step 1 (the accelerated oxidative aging):** Under N2 atmosphere, ~100 mg of sorbent was charged into tube no. 4 and mounted on the apparatus. Tubes no. 4 and no. 5 were set to 313 K. Pure water was placed in the humidification tubes (no. 1 and no. 2), and tubes no. 1, no. 2, and no. 3 were maintained at 293 K. The mixed gas supplied from the CO2/O2/N2 MFCs was passed through the humidification tubes and directed to the CO2/H2O analyzers and the O2 sensor via a bypass line. The MFC setpoints and humidifier temperatures were adjusted to achieve 400 ppm CO2, 21% O2, and a dew point of 293 K. The temperature of the sorbent tube (no. 4) was then set to 393 K, and the CO2/O2/H2O/N2 mixture was fed to the tube to initiate oxidative aging.

**Step 2 (desorption):** After 24 h of the oxidative aging, the sorbent tube (no. 4) was heated to 413 K (for (KLeu)1[Zn(Leu)2]) or maintained at 393 K (for PEI@Silica) while supplying N₂ with a dew point of 293 K to desorb CO2 adsorbed on the sorbent. Desorption was deemed complete when the outlet CO2 concentration dropped below 5 ppm, after which the sorbent tube was cooled to 313 K.

**Step 3 (CO2 uptake measurement):** CO2 uptake was then measured as described in Step 3 in Chapter 5. After the CO2 uptake measurement, the elemental analysis for the sorbents was carried out to clarify the retention of each element (C/H/N) after the accelerated durability test. Prior to the analysis, the samples were placed *in vacuo* at 393 K to remove any gases adsorbed in the sorbents.

# 7. Additional Figures and Tables


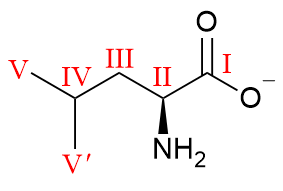

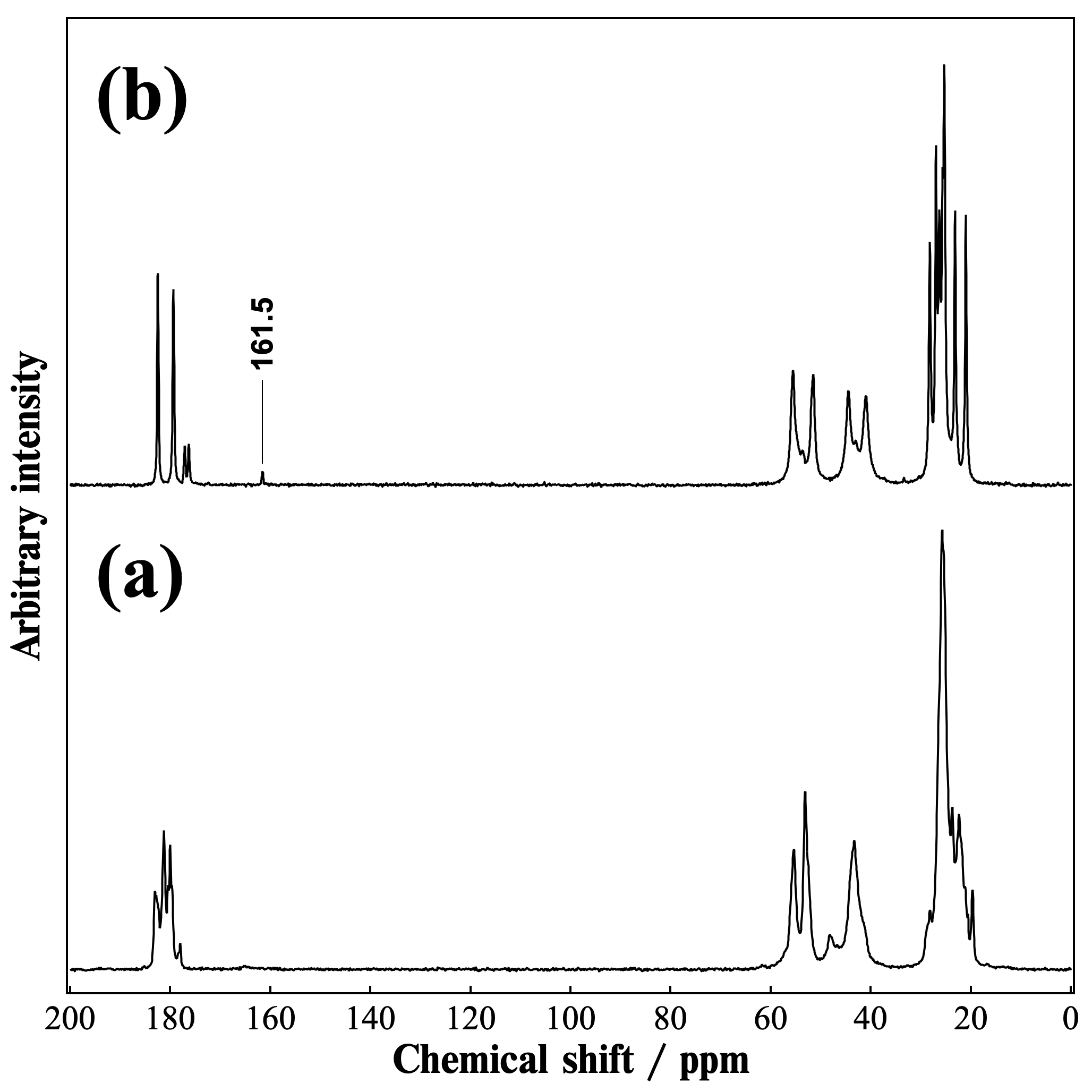


**V**

**V**’

**IV**

**II**

**III**

**I**

**Figure S1**. {1H}–13C CP/MAS NMR spectra of (a) as-synthesized (KLeu)1Zn(Leu)2 and (b) CO2 adsorbed (KLeu)1Zn(Leu)2. In (b), the roughly two-split resonance peak indicates the existence of two independent local environments of [Leu]−. A small peak observed at 161.5 ppm indicates the formation of a bicarbonate.


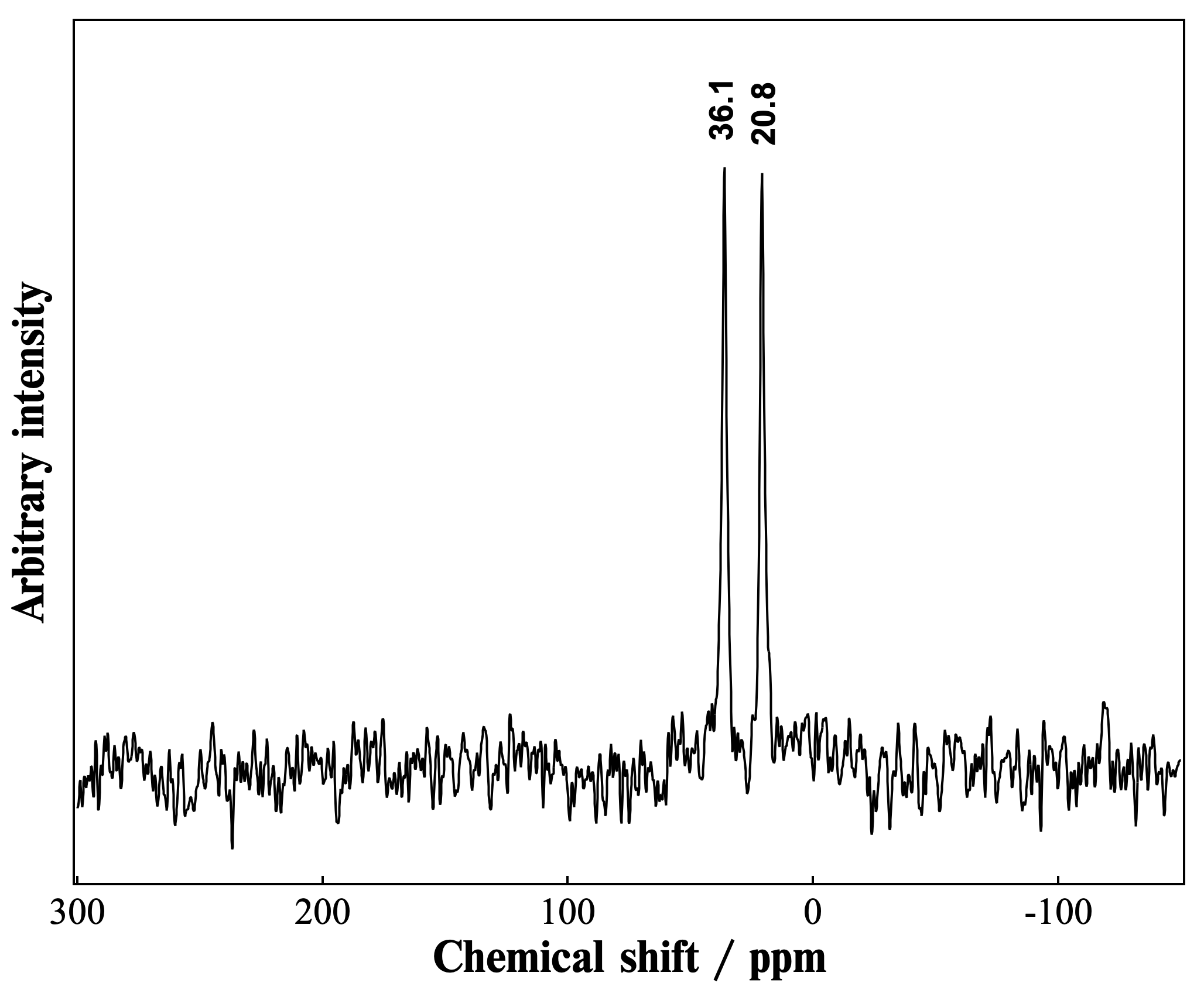


**Figure S2**. {1H}–15N CP/MAS NMR spectra of as-synthesized Zn(Leu)2. Observed two resonance peaks indicate the presence of two independent [Leu]− site in the crystal structure of Zn(Leu)2.

**(A)**

**(B)**

**
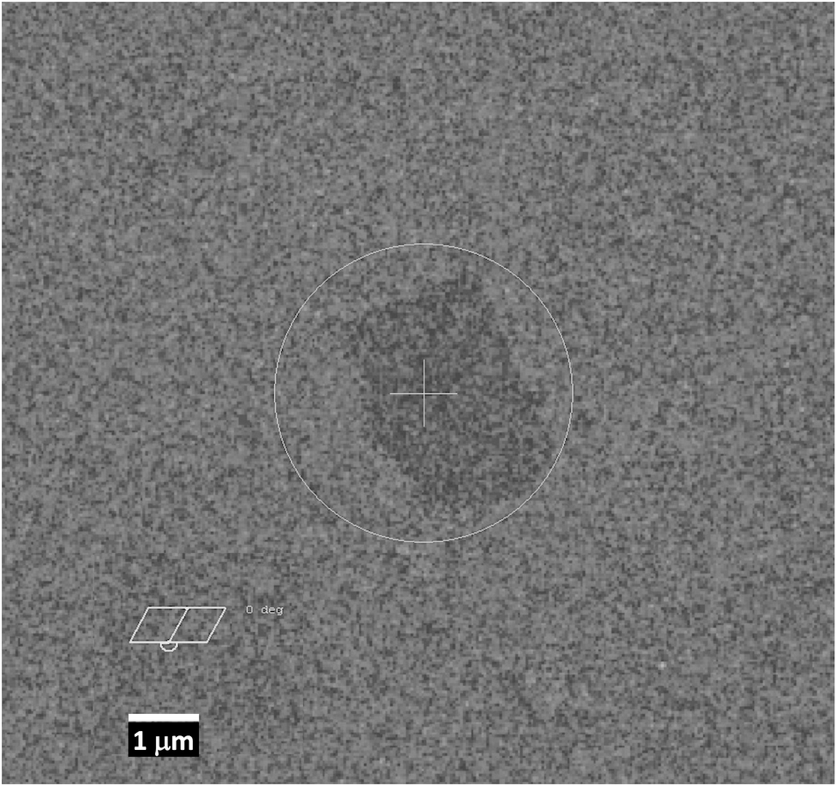
**

**
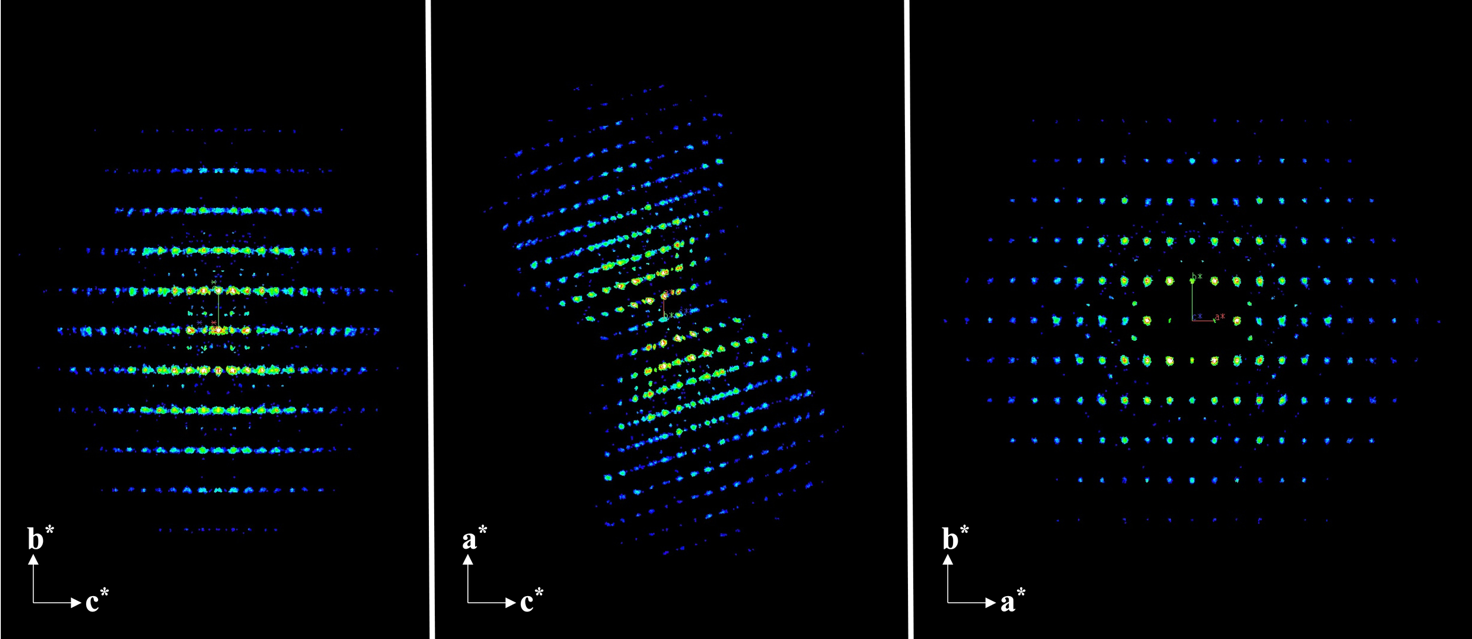
**

**Figure S3**. 3D-ED analysis using (A) a Zn(Leu)2 microcrystallite with a long axis of 3 μm and thickness < 500 nm. (B) Reciprocal 3D-ED maps viewed along *a**-, *b**-, and *c**-axes from the left side.


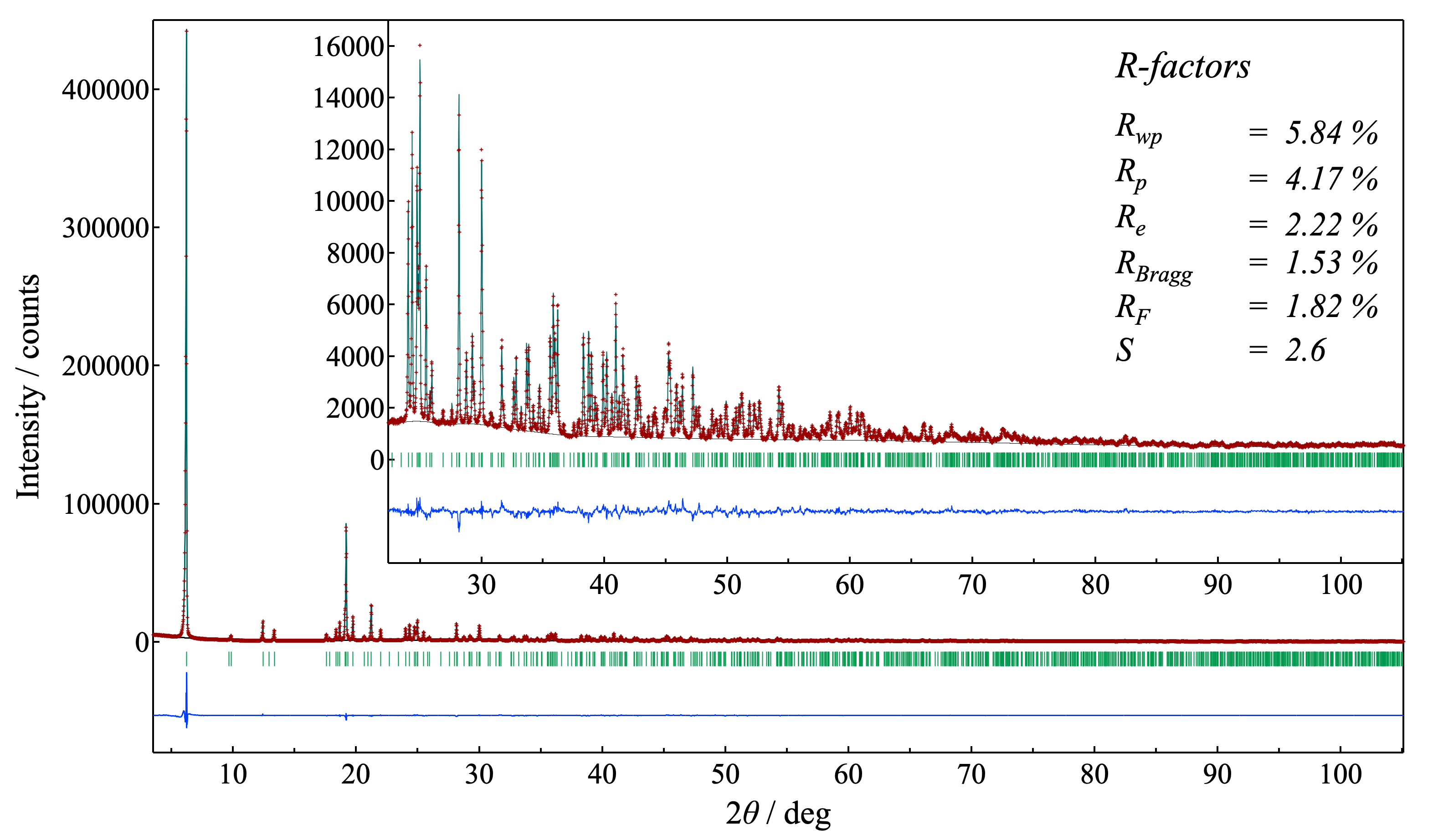


**Figure S4**. Observed (red), calculated (dark blue), and diﬀerence (blue) patterns resulting from the Rietveld analysis of Zn(Leu)2. Green vertical bars denote positions of Bragg reﬂections. The inset figure is an enlarged plot of the region where 2*θ* > 22.4°.

**
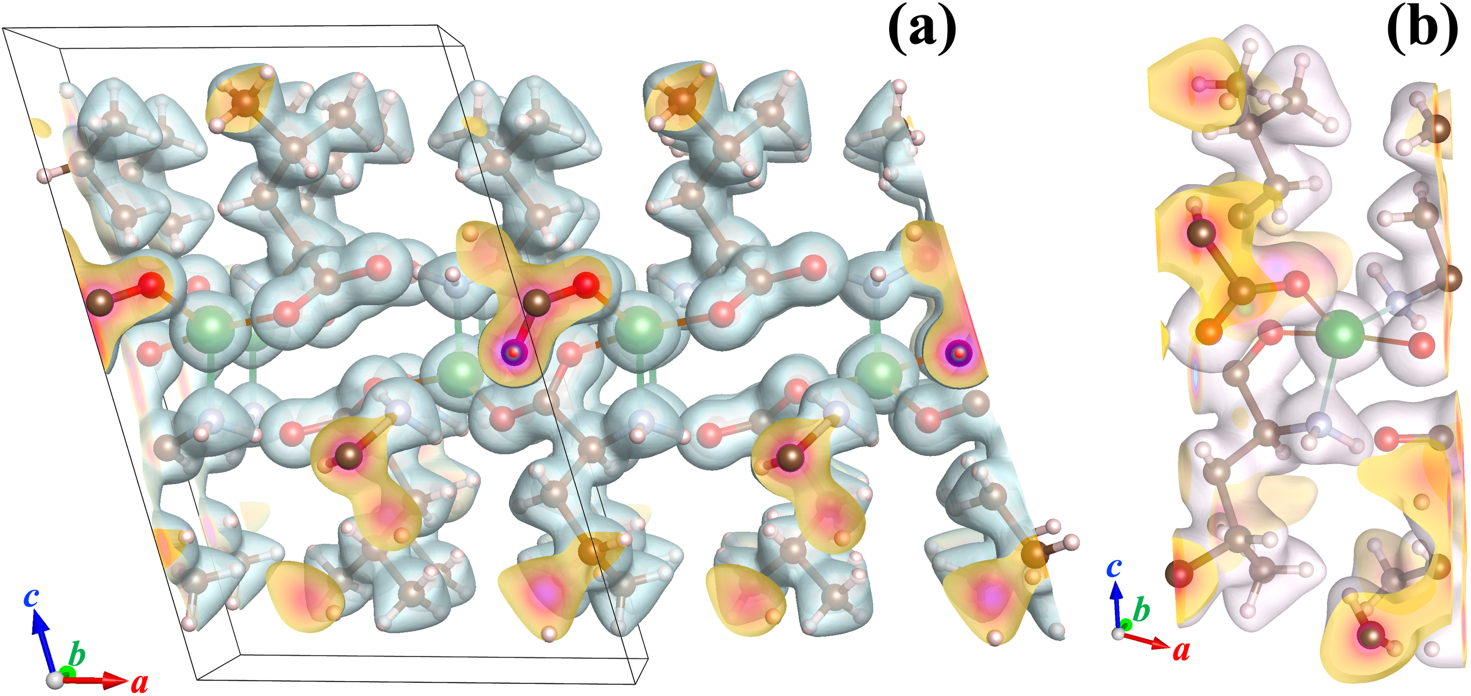
**

**Figure S5**. 3D electron density distribution (EDD) images of Zn(Leu)2 obtained by the MEM analysis. (a) EDD viewed along the *b*-axis with an isosurface level of 0.7 e/Å3. No electron density inconsistent with the structural model was observed. (b) EDD shows part of (a) with an isosurface level of 0.45 e/Å3. Electron densities observed between Zn atom and O or N atom Zn atoms indicate the formation of weak covalent bonds.


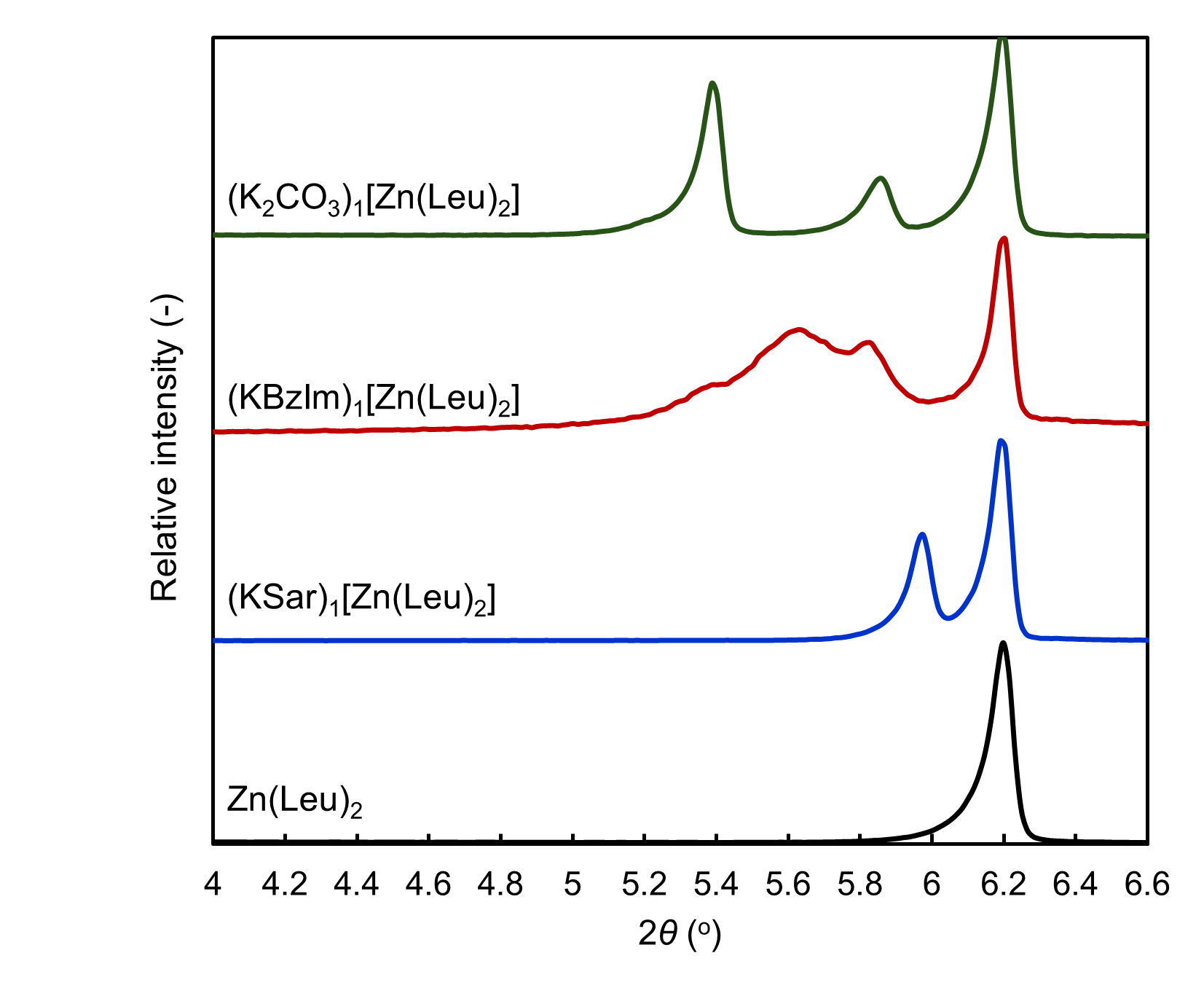


**Figure S6**. Low-angle PXRD patterns of Zn(Leu)2 (black), (KSar)1[Zn(Leu)2] (blue), (KBzIm)1[Zn(Leu)2] (red), and (K2CO3)1[Zn(Leu)2] (green).


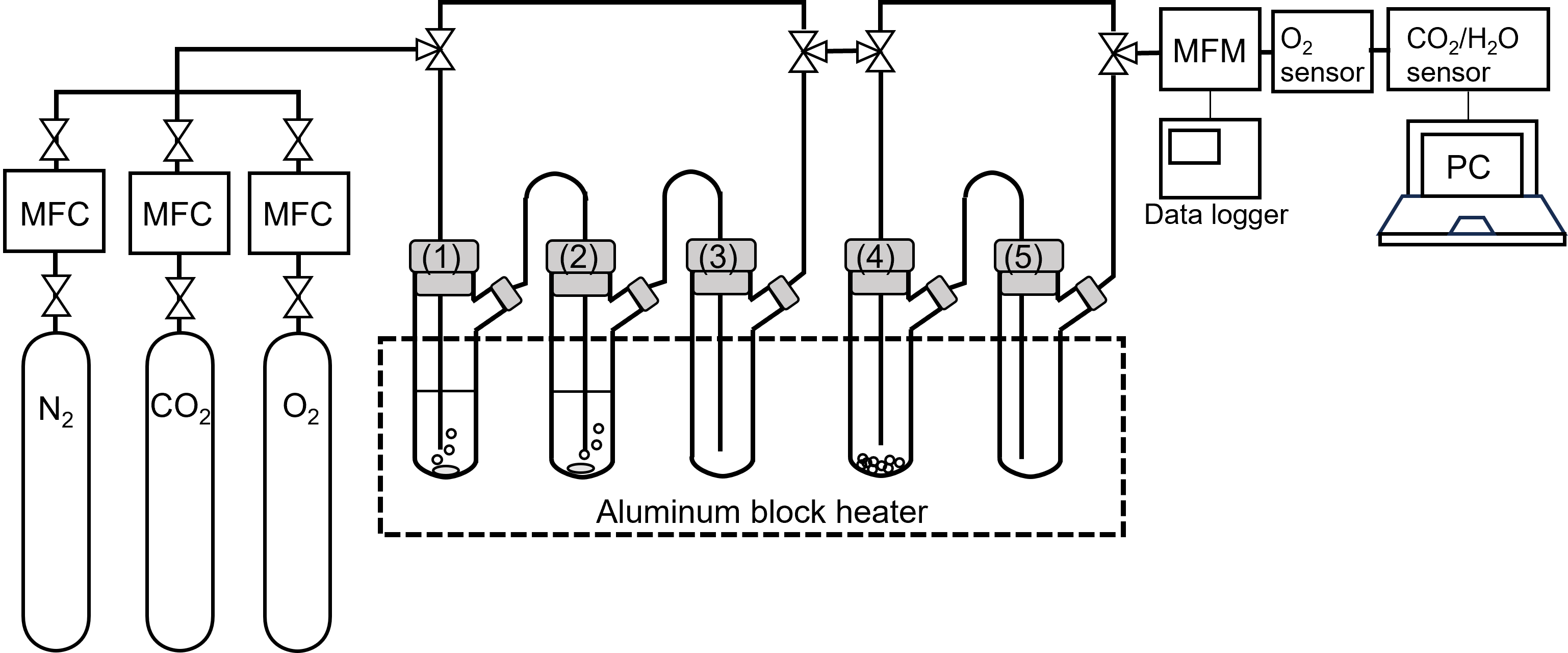


**Figure S7**. A schematic illustration of the CO2 uptake apparatus.


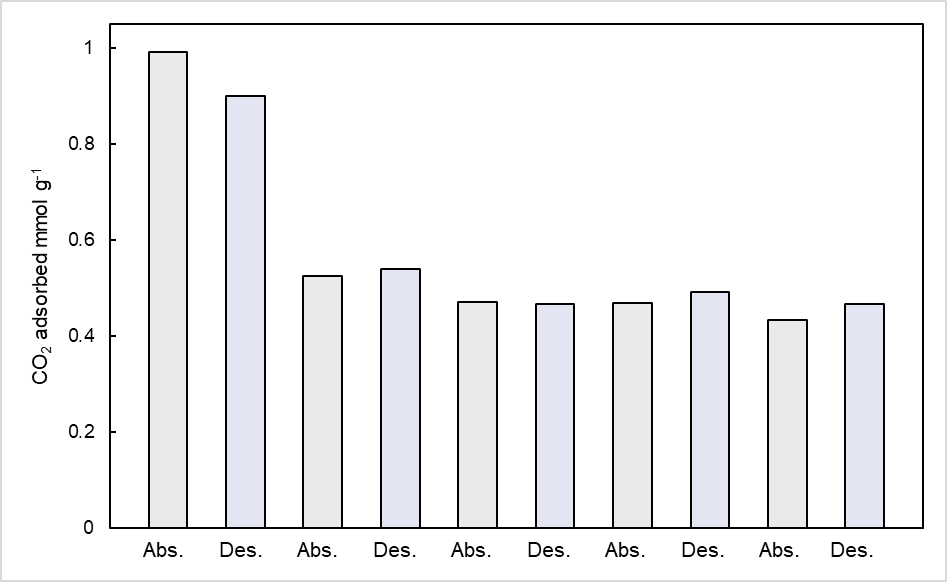


**Figure S8**. CO2 uptake values for repeated adsorption-desorption cycles of (KLeu)1[Zn(Leu)2].


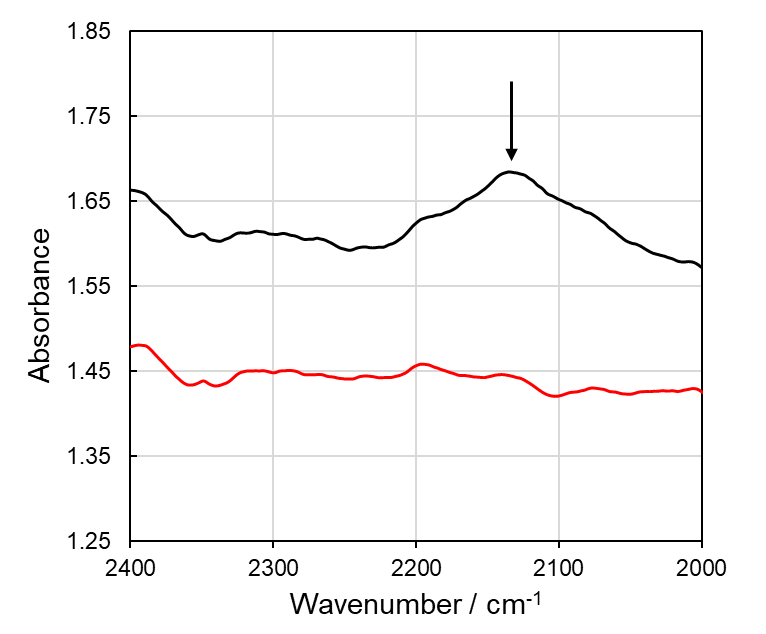

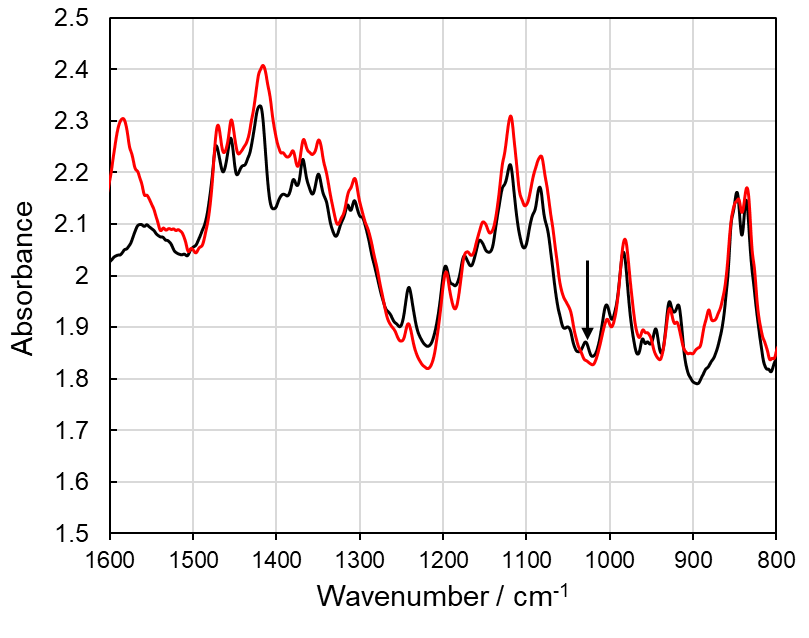


**Figure S9**. DRIFT spectra of (KLeu)1[Zn(Leu)2] after the first CO2 adsorption (black) and subsequent CO2 desorption at 393 K *in vacuo* (red). Left: region highlighting NH3+ asymmetric bending and twisting vibrations (black arrow indicates the peak). Right: region highlighting bending vibrations of COH group in bicarbonate. Arrows indicate the corresponding peaks.


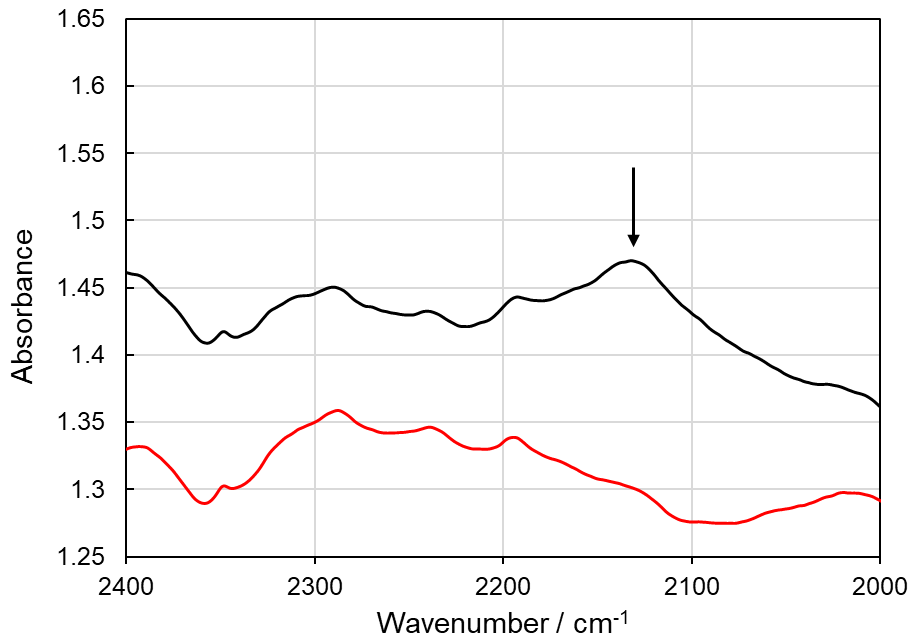

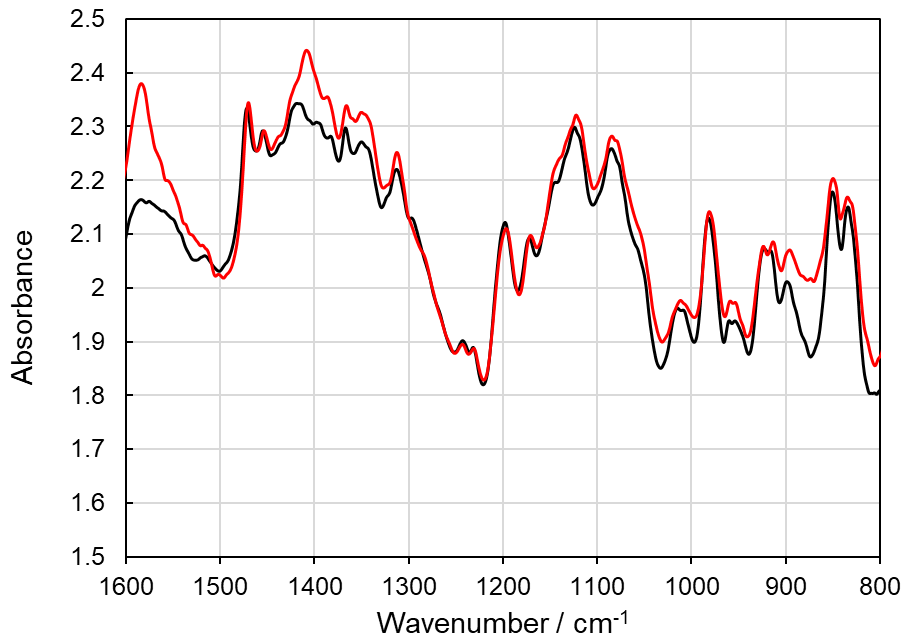


**Figure S10**. DRIFT spectra of (KLeu)1[Zn(Leu)2] after the second CO2 adsorption (black) and subsequent CO2 desorption at 393 K *in vacuo* (red).


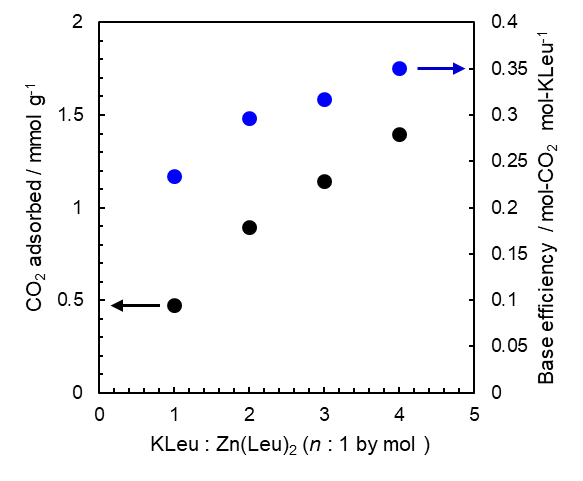


**Figure S11**. Effect of KLeu loading on CO2 uptake for (KLeu)*n*[Zn(Leu)2] (*n* = 1–4). Black: gravimetric CO2 uptake (mmol g-1). Blue: molar amount of CO2 adsorbed per mol of KLeu (“base efficiency”, mol-CO2 mol-KLeu-1)


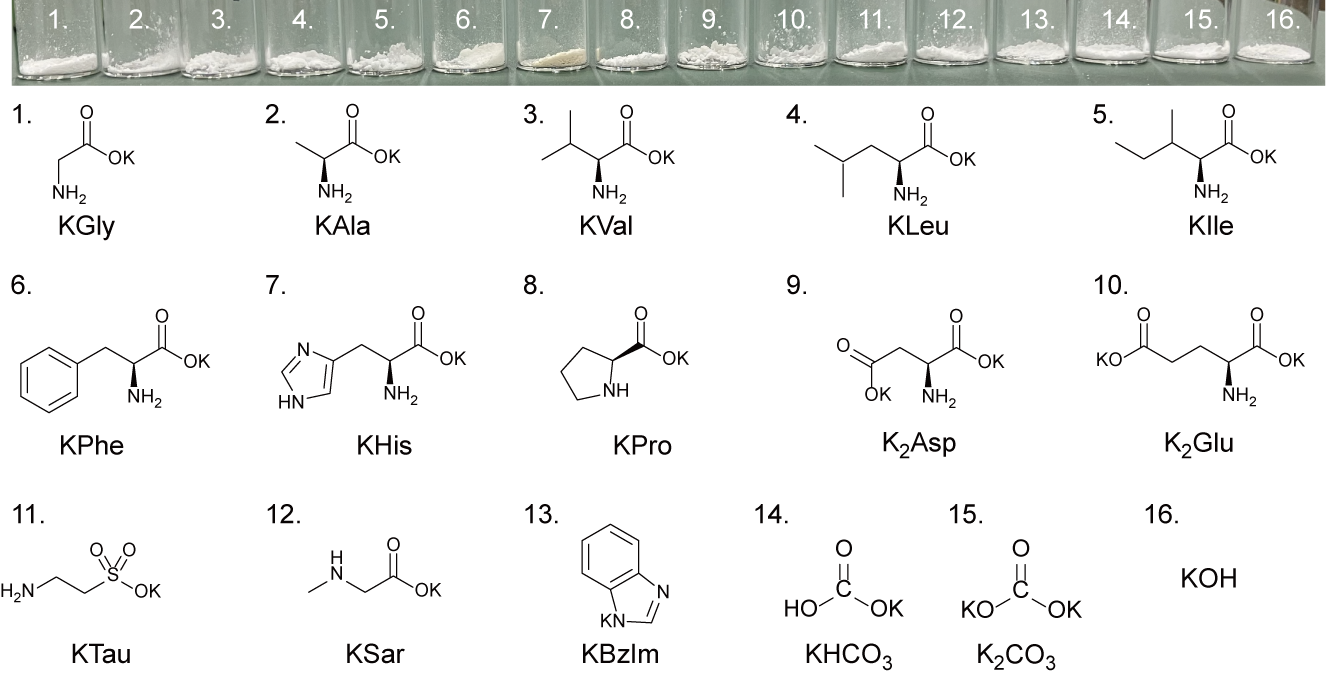


**Figure S12**. Photographs of Zn(Leu)2-supported adsorbents impregnated with the basic salts (1-16) after exposure to simulated air (400 ppm CO2; 293 K dew point; 313 K).


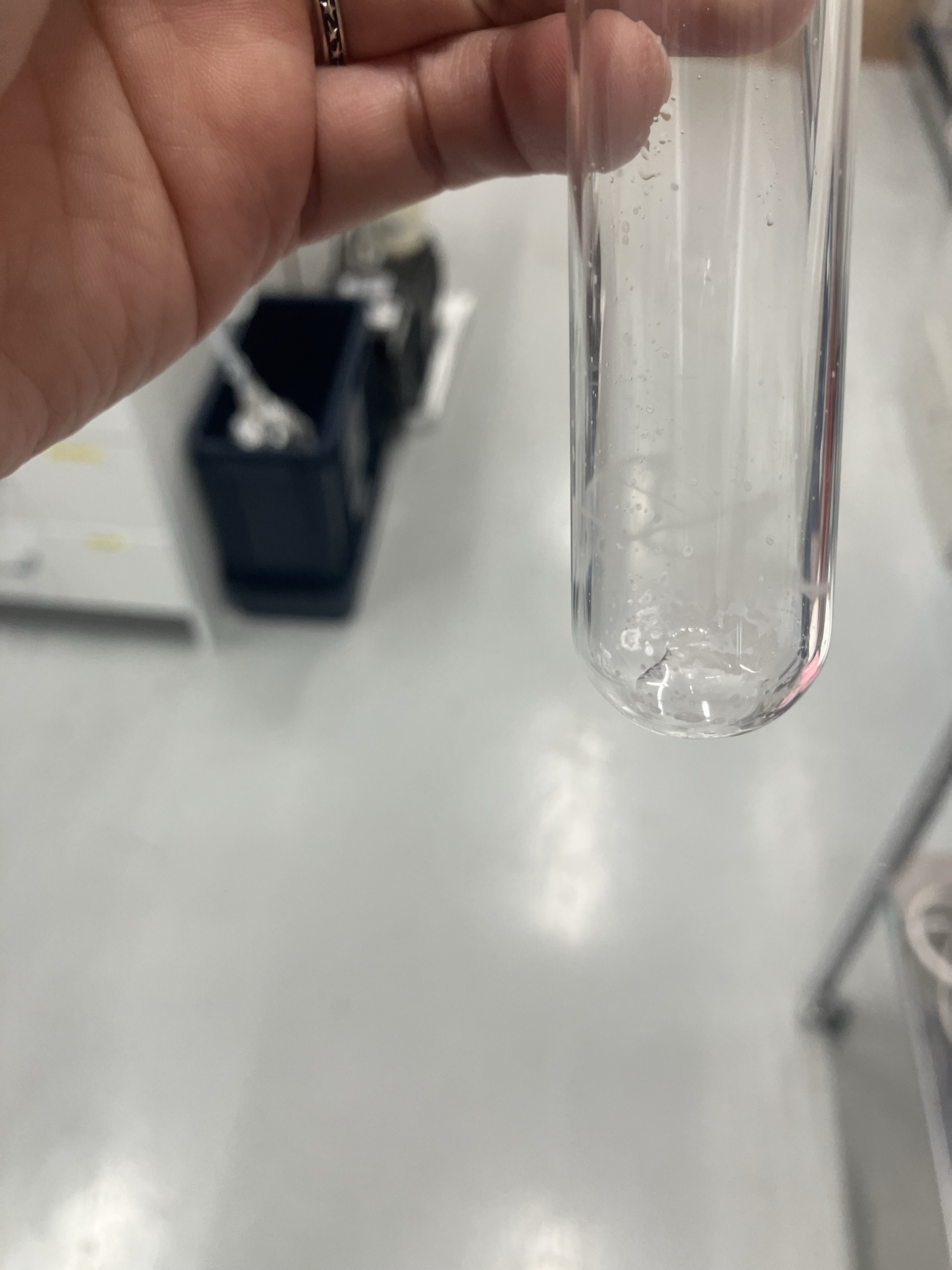

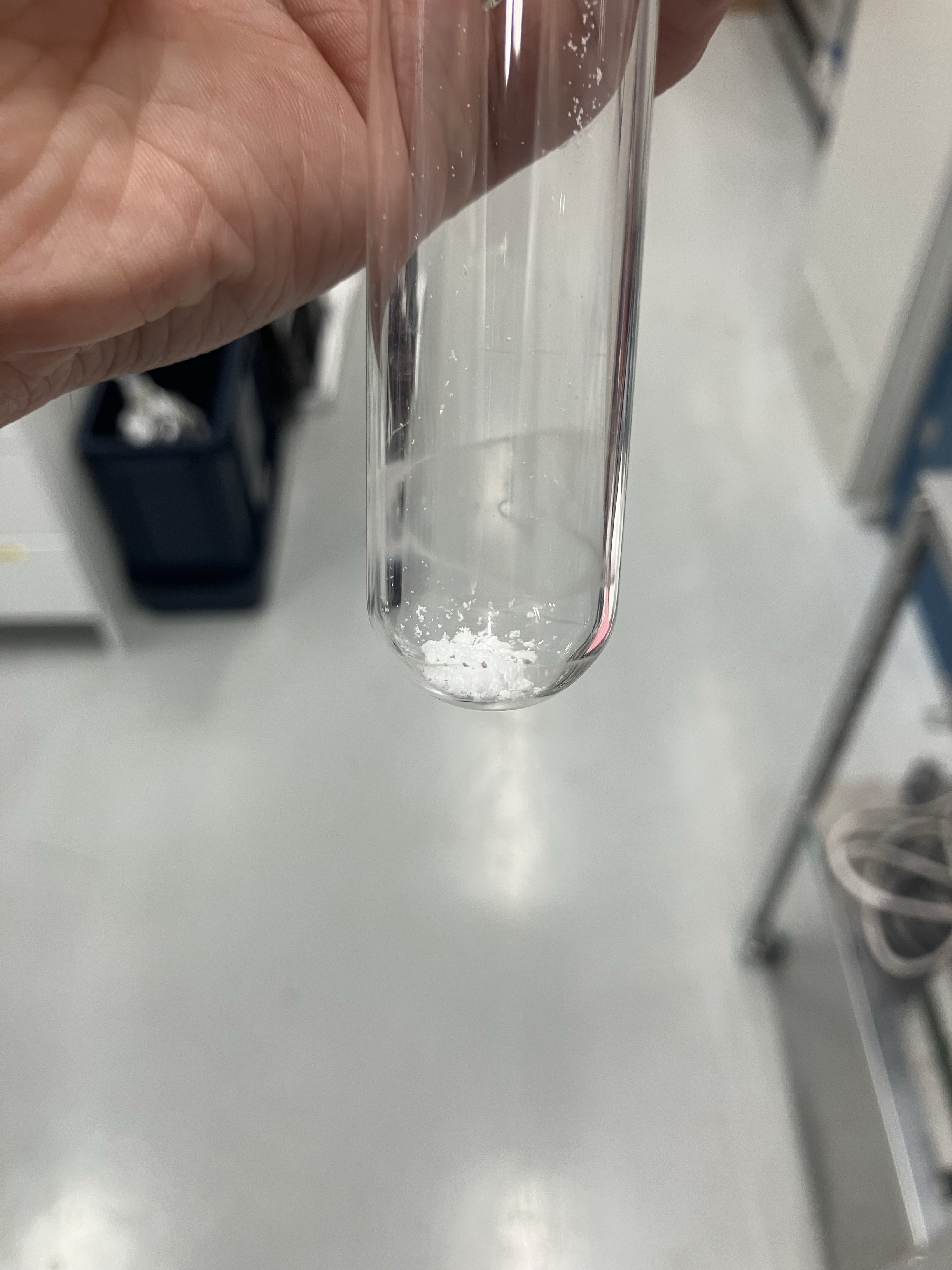


Humidified gas

CO2: 400 ppm

Dew point: 293 K

**Figure S13**. Photographs of KLeu before and after humidified gas exposure.


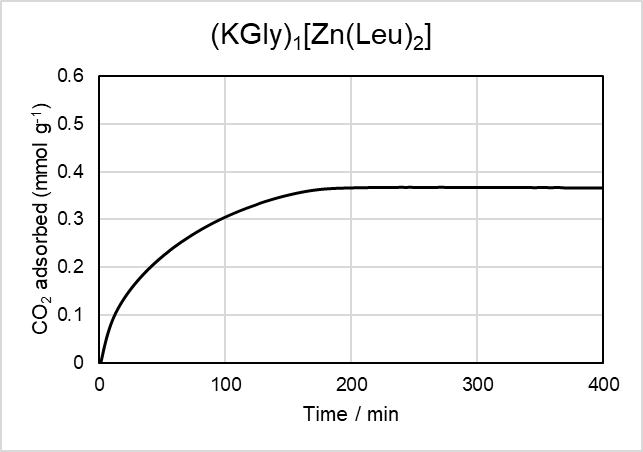


**Figure S14**. Time courses of relative CO2 uptake values for (KGly)1[Zn(Leu)2].


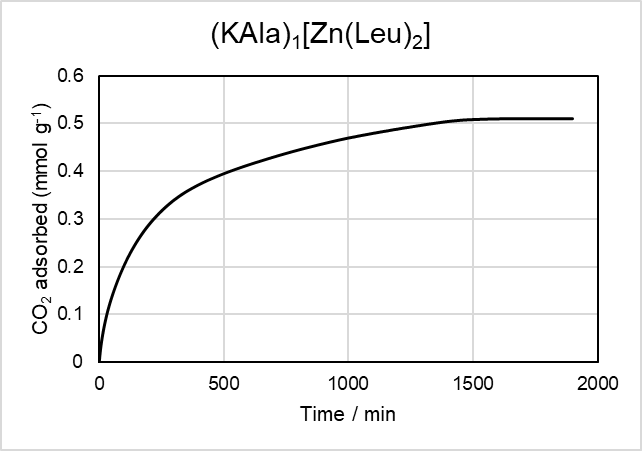


**Figure S15**. Time courses of relative CO2 uptake values for (KAla)1[Zn(Leu)2].


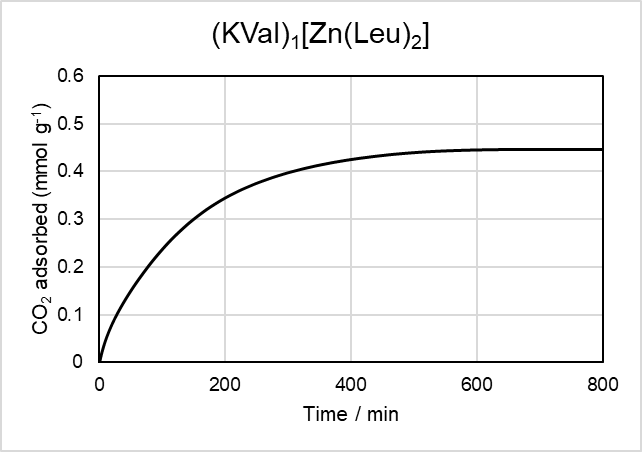


**Figure S16**. Time courses of relative CO2 uptake values for (KVal)1[Zn(Leu)2].


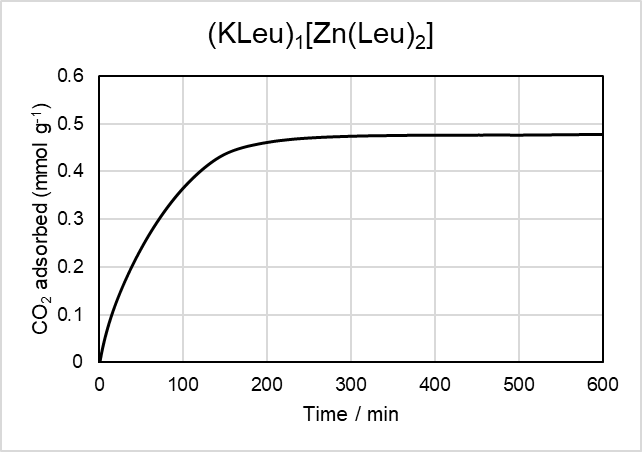


**Figure S17**. Time courses of relative CO2 uptake values for (KLeu)1[Zn(Leu)2].


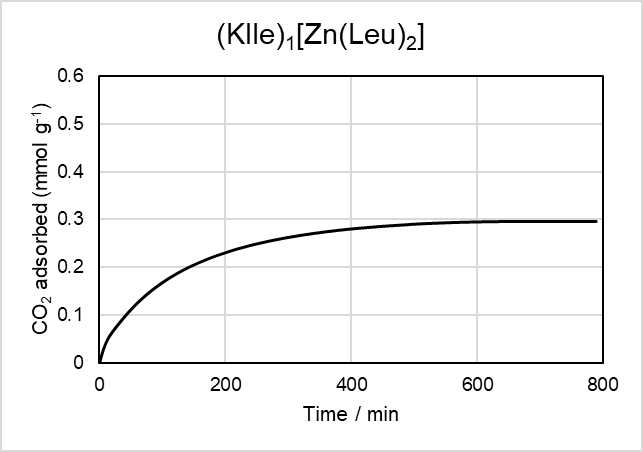


**Figure S18**. Time courses of relative CO2 uptake values for (KIle)1[Zn(Leu)2].


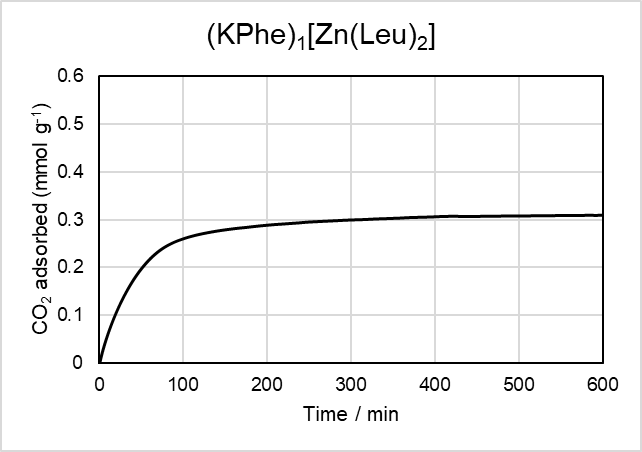


**Figure S19**. Time courses of relative CO2 uptake values for (KPhe)1[Zn(Leu)2].


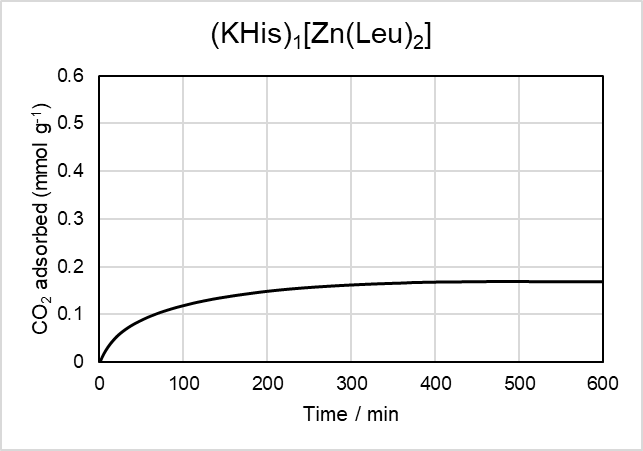


**Figure S20**. Time courses of relative CO2 uptake values for (KHis)1[Zn(Leu)2].


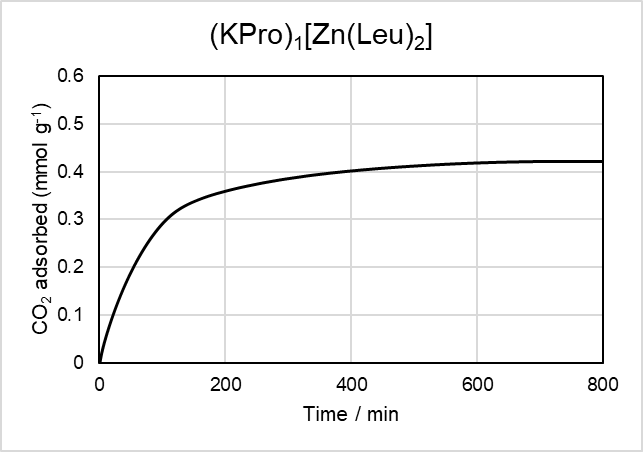


**Figure S21**. Time courses of relative CO2 uptake values for (KPro)1[Zn(Leu)2].


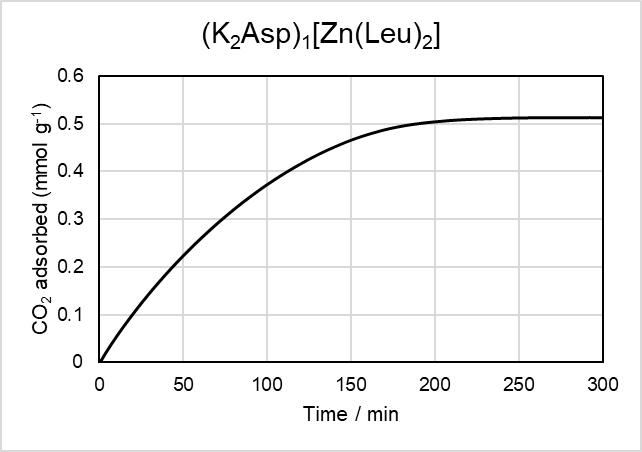


**Figure S22**. Time courses of relative CO2 uptake values for (K2Asp)1[Zn(Leu)2].


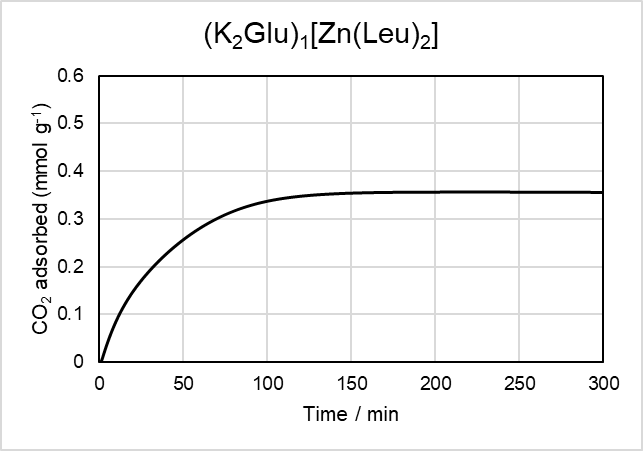


**Figure S23**. Time courses of relative CO2 uptake values for (K2Glu)1[Zn(Leu)2].


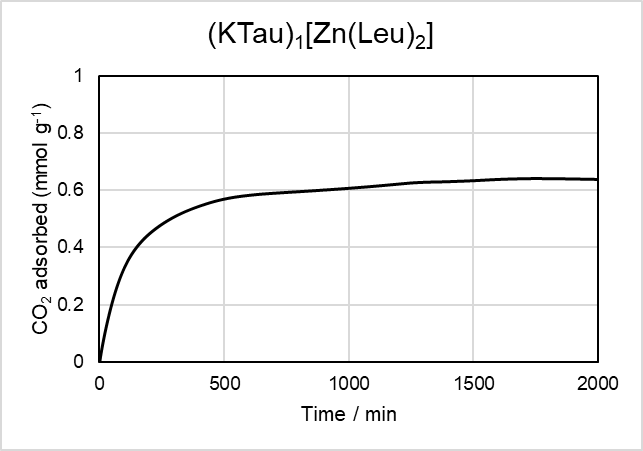


**Figure S24**. Time courses of relative CO2 uptake values for (KTau)1[Zn(Leu)2].


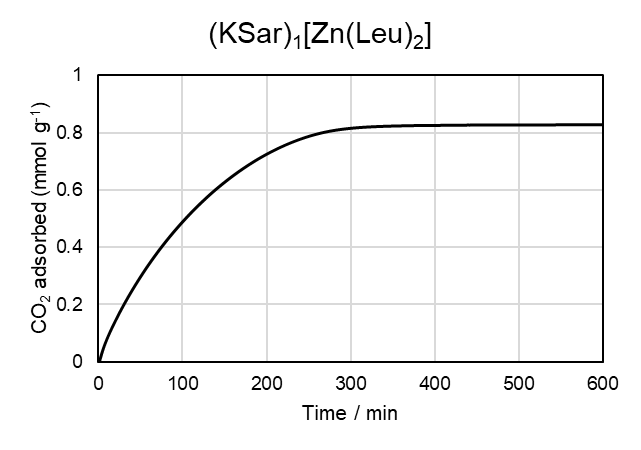


**Figure S25**. Time courses of relative CO2 uptake values for (KSar)1[Zn(Leu)2].


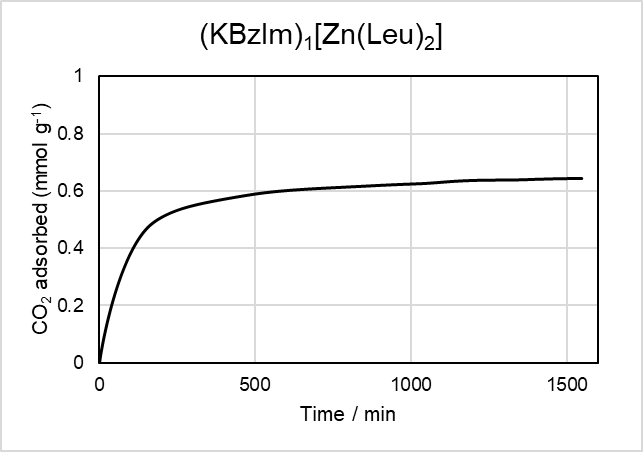


**Figure S26**. Time courses of relative CO2 uptake values for (KBzIm)1[Zn(Leu)2].


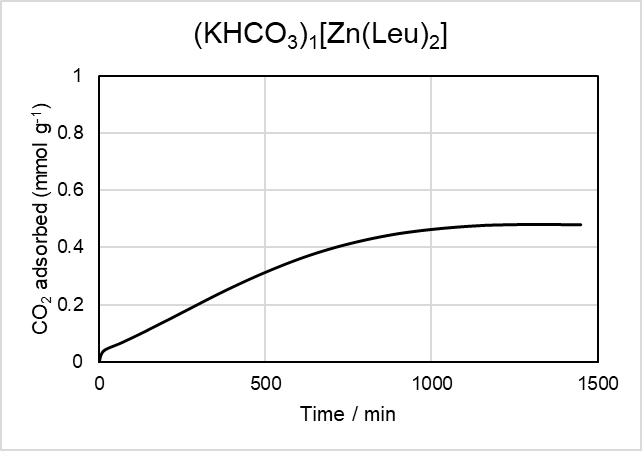


**Figure S27**. Time courses of relative CO2 uptake values for (KHCO3)1[Zn(Leu)2].


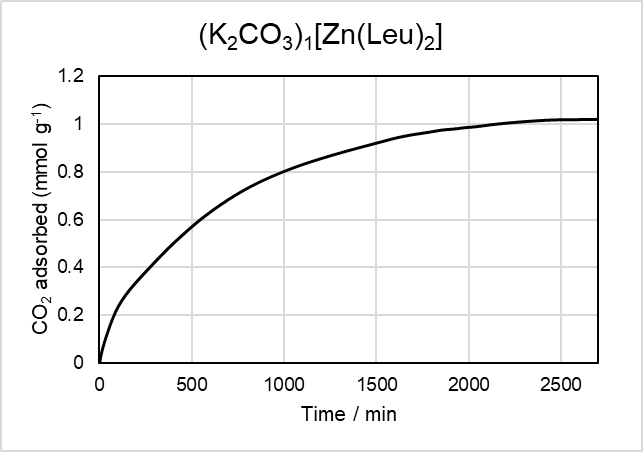


**Figure S28**. Time courses of relative CO2 uptake values for (K2CO3)1[Zn(Leu)2].


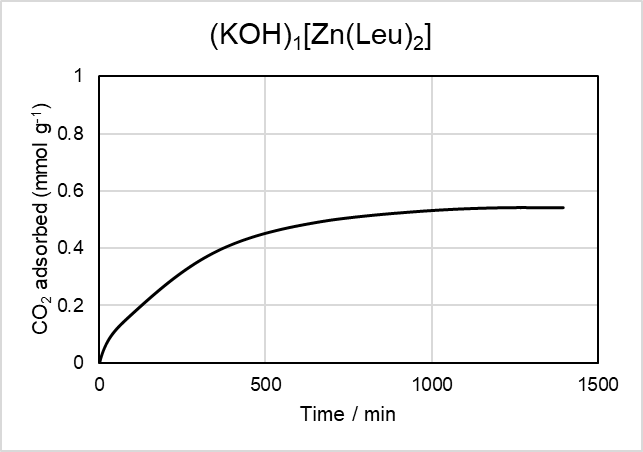


**Figure S29**. Time courses of relative CO2 uptake values for (KOH)1[Zn(Leu)2].


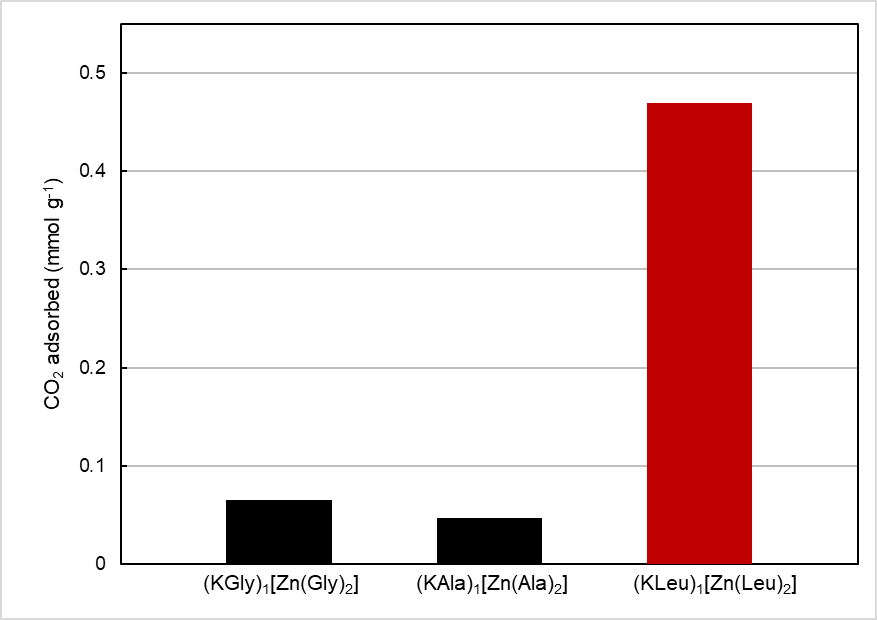


**Figure S30**. CO2 uptake for sorbents using various amino acid-based coordination polymer supports.


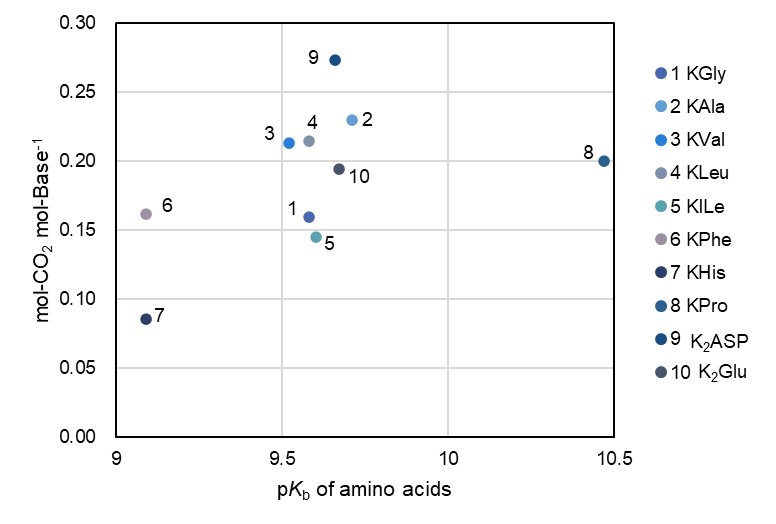


Figure S31. Relation between pKb of amino acids [s11] and base efficiency (mol-CO2 mol-base-1).


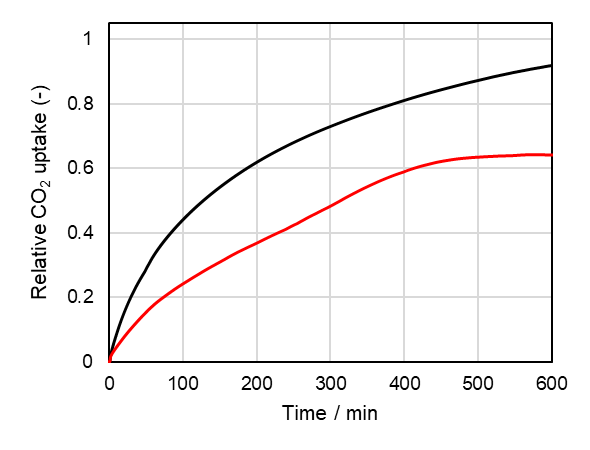


Figure S32. Time course of relative CO2 uptake values for KLeu before (black) and after (red) the accelerated oxidative aging. An aqueous solution of KLeu (30 wt%) was used for the experiment. Since water content can change during the experiment, the CO2 uptake value was calculated as the mmol of CO2 per grams of KLeu initially charged.

**Table S1.** Crystallographic data from reconstructed electron diffraction volume and experimental details for structure solution of Zn(Leu)2.

–––––––––––––––––––––––––––––––––––––––––––––––––––––––––

Crystallographic data obtained from 3D-ED data

–––––––––––––––––––––––––––––––––––––––––––––––––––––––––

Materials Zn(Leu)2

Crystal system Monoclinic

Formula C12H24N2O4Zn

*Z* 2

*Mr*325.71

Space group *P*21 (No. 4)

*a* / Å 9.8(2)

*b* / Å 5.49(18)

*c* / Å 15.2(4)

*α*  / ° 90

*β* / ° 106.7(12)

*γ* / ° 90

Temperature (K) 298

Experiments and structure solution details

–––––––––––––––––––––––––––––––––––––––––––––––––––––––––

Radiation, l / Å 0.0251

Tilt range / ° –40 / +60

Tilt step / ° 0.5

Number of total reflections 3365

Number of independent reflections 2102

The limits on the Miller indices –12 < *h* < 12

–6 < *k* < 6

–18 < *l* < 18

Completeness / % 66.4

Resolution / Å 0.91

Number of parameters 131

*R*int 0.145

w*R*(*F*2) 0.308

*R*1 (*I* > 2 σ(*I*)) 0.145

–––––––––––––––––––––––––––––––––––––––––––––––––––––––––

**Table S2.** Obtained crystallographic information of Zn(Leu)2 by the Rietveld refinement.

––––––––––––––––––––––––––––––––––––––––––––––––––––––––––––––

Sample name Zn(Leu)2

––––––––––––––––––––––––––––––––––––––––––––––––––––––––––––––

Refined structural composition C12H24N2O4Zn

*F*W 325.71

Crystal system Monoclinic

Space group *P*21 (No. 4)

*a* / Å 9.5918(2)

*b* / Å 5.39286(11)

*c* / Å 14.8963(4)

*b* / ° 106.831(2)

Unit-cell volume, *V* / Å3 737.53(3)

Wavelength, *λ* / nm 0.1540593 (Cu Kα1)

2*θ* range/° 3.0–105.1

Step size, 2*θ* / ° 0.0163

Profile range in the unit of FWHM 12

FWHM / ° (at 2*θ* = 6.194°) 0.072

Number of intensity data 6258

Number of contributing reflections 963

Number of refined structural parameters 136

Number of background parameters 12

Number of nonlinear restraints 108

Reliability indexes

*RF* 0.018

*R*Bragg 0.015

*R*wp 0.058

*R*p 0.042

*R*exp 0.022

*S* 2.22

χ2 4.92

––––––––––––––––––––––––––––––––––––––––––––––––––––––––––––––

**Table S3.** Crystallographic Information File (CIF) of Zn(Leu)2.

#========================================================================

# CRYSTAL DATA

#---------------------------------------------------------------------------------------------------------------------

data_VESTA_phase_1

_chemical_name_common 'Zn(Leu)2'

_cell_length_a 9.59175(14)

_cell_length_b 5.39286(5)

_cell_length_c 14.89630(14)

_cell_angle_alpha 90.000000

_cell_angle_beta 106.8310(11)

_cell_angle_gamma 90.000000

_cell_volume 737.532743

_space_group_name_H-M_alt 'P 21'

_space_group_IT_number 4

loop_

_space_group_symop_operation_xyz

'x, y, z'

'-x, y+1/2, -z'

loop_

_atom_site_label

_atom_site_occupancy

_atom_site_fract_x

_atom_site_fract_y

_atom_site_fract_z

_atom_site_adp_type

_atom_site_B_iso_or_equiv

_atom_site_type_symbol

Zn 1.0 0.2390(5) 0.319530 0.5363(3) Biso 2.797000 Zn

Oa1 1.0 0.1562(18) 0.093(3) 0.6052(11) Biso 2.542000 O

Oa2 1.0 0.9483(18) 0.048(4) 0.5025(14) Biso 2.542000 O

Na3 1.0 0.818(3) 0.768(5) 0.6068(12) Biso 2.699000 N

Ca4 1.0 0.015(5) 0.628(8) 0.803(3) Biso 5.461000 C

Ca5 1.0 0.067(4) 0.725(7) 0.717(3) Biso 5.461000 C

Ca6 1.0 -0.042(4) 0.906(7) 0.651(3) Biso 5.461000 C

Ca7 1.0 -0.023(4) 0.830(10) 0.859(3) Biso 5.461000 C

Ca8 1.0 0.142(5) 0.461(7) 0.863(3) Biso 5.461000 C

Ca9 1.0 0.035(5) 0.003(7) 0.5778(18) Biso 5.461000 C

Ha10 1.0 0.93(3) 0.52(5) 0.781(19) Biso 5.000000 H

Ha11 1.0 0.08(4) 0.59(4) 0.679(19) Biso 5.000000 H

Ha12 1.0 0.16(2) 0.80(8) 0.737(16) Biso 5.000000 H

Ha13 1.0 0.93(3) 0.04(6) 0.68(2) Biso 5.000000 H

Ha14 1.0 0.87(3) 0.86(7) 0.842(15) Biso 5.000000 H

Ha15 1.0 0.01(3) 0.78(7) 0.924(12) Biso 5.000000 H

Ha16 1.0 0.03(4) 0.98(6) 0.847(18) Biso 5.000000 H

Ha17 1.0 0.12(3) 0.29(8) 0.858(17) Biso 5.000000 H

Ha18 1.0 0.16(3) 0.51(5) 0.925(15) Biso 5.000000 H

Ha19 1.0 0.23(3) 0.47(5) 0.842(16) Biso 5.000000 H

Ha20 1.0 0.73(3) 0.84(6) 0.629(15) Biso 5.000000 H

Ha21 1.0 0.83(3) 0.58(5) 0.624(16) Biso 5.000000 H

Ob1 1.0 0.4431(18) 0.164(4) 0.5608(10) Biso 2.542000 O

Ob2 1.0 0.6829(17) 0.232(3) 0.6323(10) Biso 2.542000 O

Nb3 1.0 0.365(3) 0.646(4) 0.5911(16) Biso 2.699000 N

Cb4 1.0 0.567(5) 0.247(7) 0.816(3) Biso 5.461000 C

Cb5 1.0 0.451(4) 0.430(8) 0.745(3) Biso 5.461000 C

Cb6 1.0 0.498(5) 0.519(8) 0.656(4) Biso 5.461000 C

Cb7 1.0 0.692(4) 0.427(7) 0.874(3) Biso 5.461000 C

Cb8 1.0 0.499(5) 0.106(7) 0.885(4) Biso 5.461000 C

Cb9 1.0 0.549(3) 0.297(8) 0.6113(18) Biso 5.461000 C

Hb10 1.0 0.60(4) 0.12(5) 0.78(3) Biso 5.000000 H

Hb11 1.0 0.36(3) 0.34(7) 0.727(14) Biso 5.000000 H

Hb12 1.0 0.44(4) 0.58(5) 0.78(2) Biso 5.000000 H

Hb13 1.0 0.57(3) 0.65(4) 0.67(2) Biso 5.000000 H

Hb14 1.0 0.64(3) 0.56(7) 0.90(2) Biso 5.000000 H

Hb15 1.0 0.74(3) 0.49(5) 0.830(19) Biso 5.000000 H

Hb16 1.0 0.76(3) 0.34(8) 0.926(14) Biso 5.000000 H

Hb17 1.0 0.43(3) -0.01(5) 0.848(19) Biso 5.000000 H

Hb18 1.0 0.46(3) 0.22(5) 0.920(15) Biso 5.000000 H

Hb19 1.0 0.58(4) 0.01(5) 0.927(18) Biso 5.000000 H

Hb20 1.0 0.407(19) 0.79(7) 0.562(14) Biso 5.000000 H

Hb21 1.0 0.30(3) 0.73(4) 0.630(15) Biso 5.000000 H

#========================================================================

**Table S4.** CO2 uptake for (KLeu)*n*[Zn(Leu)2] and that of literature values for PEI-impregnated solid sorbents.

| Base | Support | Loading amount  /mmol-N g-1 | Temp,  /K | CO2  /ppm | Dew point  /K | CO2 adsorbed  /mmol g-1 | Ref. |
| --- | --- | --- | --- | --- | --- | --- | --- |
| PEI | Silica | 8.4 | 313 | 400 | 293 | 1.11 | This study |
| KLeu | Zn(Leu)2 | 2.0 | 313 | 400 | 293 | 0.47 | This study |
| KLeu | Zn(Leu)2 | 4.0 | 313 | 400 | 293 | 1.40 | This study |
| PEI | g-Alumina | 13.1 | 303 | 400 | Dry | 1.05 | [s12] |
| PEI | g-Alumina | 11.2 | 298 | 400 | Dry | 1.74 | [s13] |
| PEI | SBA-15 | 9.2 | 298 | 400 | Dry | 1.05 | [s13] |
| PEI | Zr-SBA-15 | 8.3 | 298 | 400 | Dry | 0.85 | [s14] |
| PEI | Mesocellular foam silica | 10.7 | 298 | 400 | Dry | 1.74 | [s15] |
| PEI | Fumed silica | (33%) | 298 | 420 | Dry | 1.18 | [s16] |
| PEI | Fumed silica | (33%) | 298 | 420 | 291 | 1.77 | [s16] |
| PEI | Fumed silica | (55%) | 298 | 420 | Dry | 1.71 | [s16] |
| PEI | Fumed silica | (55%) | 298 | 420 | 293 | 1.41 | [s16] |

# 8. References in Supporting Information

[s1] S. Ito, F. J. White, E. Okunishi, Y. Aoyama, A. Yamano, H. Sato, J.D. Ferrara, M. Jasnowskie and M. Meyer, *CrystEngComm.* **2021**, *23*, 8622–8630.

[s2] G. M. Sheldrick, *Acta Crystallogr*. *A* **2008**, *64*, 112–122.

[s3] G. M. Sheldrick, *Acta Crystallogr*. *C* **2015**, *71*, 3–8.

[s4] O. V. Dolomanov, L. J. Bourhis, R. J. Gildea, J. A. K. Howarda, H Puschmanna, *J. Appl. Crystallogr.* **2009**, *42*, 339–341.

[s5] R. Oishi-Tomiyasu, *J. Appl. Cryst.* **2014**, *47* 2055–2059.

[s6] F. Izumi, K. Momma, *Solid State Phenom*. **2007**, *130*, 15–20.

[s7] M. Takata, *Acta* *Crystallogr*. *A* **2008**, *64*, 232–245.

[s8] K. Momma, T. Ikeda, A. A. Belik, F. Izumi, *Powder Diffr.* **2013**, *28*, 184–193.

[s9] K. Momma, F. Izumi, *J*. *Appl*. *Crystallogr*. **2011**, *44*, 1272–1276.

[s10] F. Izumi, T. Ikeda, *Mater*. *Sci*. *Forum* **2000**, *198*, 321–324.

[s11] J. R. Rumble (Ed.), *CRC Handbook of Chemistry and Physics*, 102nd ed., CRC Press, 2021.

[s12] J. S. A. Carneiro, G. Innocenti, H. J. Moon, Y. Guta, L. Proaño, C. Sievers, M. A. Sakwa-Novak, E. W. Ping, C. W. Jones, *Angew. Chem. Int. Ed.* **2023**, *62*, e202302887.

[s13] W. Chaikittisilp, H.-J. Kim, C. W. Jones, *Energy Fuels* **2011**, *25*, 5528–5537.

[s14] Y. Kuwahara, D.-Y. Kang, J. R. Copeland, N. A. Brunelli, S. A. Didas, P. Bollini, C. Sievers, T. Kamegawa, H. Yamashita, C. W. Jones, *J. Am. Chem. Soc.* **2012**, *134*, 10757–10760.

[s15] W. Chaikittisilp, R. Khunsupat, T. T. Chen, C. W. Jones, *Ind. Eng. Chem. Res.* **2011**, *50*, 14203–14210.

[s16] A. Goeppert, M. Czaun, R. B. May, G. K. S. Prakash, G. A. Olah, S. R. Narayanan, *J. Am. Chem. Soc.* **2011**, *133*, 20164–20167.
